# Exploring the Mangrove Fruit: From the Phytochemicals to Functional Food Development and the Current Progress in the Middle East

**DOI:** 10.3390/md20050303

**Published:** 2022-04-28

**Authors:** Fitri Budiyanto, Eman A. Alhomaidi, Afrah E. Mohammed, Mohamed A. Ghandourah, Hajer S. Alorfi, Nahed O. Bawakid, Wailed M. Alarif

**Affiliations:** 1 Department of Marine Chemistry, Faculty of Marine Sciences, King Abdulaziz University, P.O. Box 80207, Jeddah 21589, Saudi Arabia; fitri.budiyanto@gmail.com (F.B.); mghandourah@kau.edu.sa (M.A.G.); welaref@kau.edu.sa (W.M.A.); 2 National Research and Innovation Agency, Jl. M.H. Thamrin No. 8, Jakarta 10340, Indonesia; 3 Department of Biology, Faculty of Science, Princess Nourah bint Abdulrahman University, P.O. Box 84428, Riyadh 11671, Saudi Arabia; afamohammed@pnu.edu.sa; 4 Department of Chemistry, Faculty of Science, King Abdulaziz University, P.O. Box 80203, Jeddah 21589, Saudi Arabia; halorfi@kau.edu.sa (H.S.A.); nbawaked@kau.edu.sa (N.O.B.)

**Keywords:** mangrove fruit, secondary metabolites, terpenoids, nutrition, functional food

## Abstract

Nowadays, the logarithmic production of existing well-known food materials is unable to keep up with the demand caused by the exponential growth of the human population in terms of the equality of access to food materials. Famous local food materials with treasury properties such as mangrove fruits are an excellent source to be listed as emerging food candidates with ethnomedicinal properties. Thus, this study reviews the nutrition content of several edible mangrove fruits and the innovation to improve the fruit into a highly economic food product. Within the mangrove fruit, the levels of primary metabolites such as carbohydrates, protein, and fat are acceptable for daily intake. The mangrove fruits, seeds, and endophytic fungi are rich in phenolic compounds, limonoids, and their derivatives as the compounds present a multitude of bioactivities such as antimicrobial, anticancer, and antioxidant. In the intermediary process, the flour of mangrove fruit stands as a supplementation for the existing flour with antidiabetic or antioxidant properties. The mangrove fruit is successfully transformed into many processed food products. However, limited fruits from species such as *Bruguiera gymnorrhiza, Rhizophora mucronata*, *Sonneratia caseolaris*, and *Avicennia marina* are commonly upgraded into traditional food, though many more species demonstrate ethnomedicinal properties. In the Middle East, *A*. *marina* is the dominant species, and the study of the phytochemicals and fruit development is limited. Therefore, studies on the development of mangrove fruits to functional for other mangrove species are demanding. The locally accepted mangrove fruit is coveted as an alternate food material to support the sustainable development goal of eliminating world hunger in sustainable ways.

## 1. Introduction

Prolonged drought and other natural disasters drive food shortages, and with the global COVID-19 pandemic over the last two years, the global food distribution has been left in disarray. Disruption of the food supply and poverty cause inequity in accessing nutritious food stocks [1,2]. Regarding the UN report in 2021, up to 811 million of the world’s population is threatened by undernourishment, which represents an increase from previous years, whilst the production rate and economic aspect continue to disturb the food stock and distribution [3,4]. Those massive obstacles obstruct the sustainable development program adopted by the UN, especially point 2, to ensure sustainable manufacturer and consumption patterns to negate world hunger [5]. Thus, the search for emerging alternative food sources with a nutrition balance is requested [6]. Even though it is not a staple food for many populations, the consumption of fruit is steadily growing for its health benefit, the daily intake of which can be considered useful in providing nutrition supplementation [7]. Some types of mangroves produce edible fruit. Though it is not categorized as a commonly cultivated plant, the mangrove fruit for many communities is consumed for its ethnomedicinal properties.

Mangroves are halophile plants with vital economic and ecological services [8]. The area is considered the most productive ecosystem, underlying the fisheries’ food web [9]. Mangrove areas are wood/timber producers, feeding–nesting ground for birds, and consumable fishery commodities such as fish and shellfish [10,11]. On the global scale, the mangrove area is a main natural contributor in managing the climate in complex ways, such as its carbon flux mechanism and sequestration scheme [12,13]. However, the mangrove area hides its useful function on a smaller scale, especially for human merit. The mangrove sediments are rich in nutrients due to the rapid decomposition of organic matters [14], and it holds financial worth up to USD 232.49 per hectare when transformed into fertilizer in the agroindustry sector [15,16]. However, the equilibrium between conservation and exploration is compulsory to contain the sustainability effort [17,18,19].

Various natural treasures are found, from deeply buried in the sediment to high up in the canopy within the mangrove forest. Many microorganisms as micro-producers reside within the ecosystem with their irreplaceable roles [20,21]. Mangrove plants are hosts for more than 850 fungi, while 38 are classified as endophytic symbionts [22]. Several associated bacteria are also well-recognized for synthesizing phytochemicals, such as compounds from *Pseudoalteromonas xiamenensis* for its antibiotic properties [23,24] and *Streptomyces euryhalinus* for its antioxidant properties [25]. Aside from the symbionts, all parts of mangroves have been used in folklore medicine since time immemorial [26]. The parts of *Rhizophora mangle*, *Avicennia officinalis* L., and *Xylocarpus granatum* J. Koenig are renowned for their pharmacological and ethnomedicinal usage [27,28,29]. Those bioactivities are assumed from the metabolite contents within the plant. Naphthofuranquinone with intense anti-trypanosomal activity is found in the twigs of *A. lanata* [30], while proanthocyanidins from leaves of *Ceriops tagal* show solid antioxidant activities [31]. Unfortunately, only 27 species of mangrove have been traditionally used [32]. Among parts of the mangrove, the fruit is a seducing subject for exploration. The fruit is known for its prowess in traditional medicine, such as treating asthma, bleeding, cough, febrifuge, hemorrhages, intestinal parasites, remedy piles, sprains, swelling, and ulcers [32]. Some mangrove fruits are also edible, assigning their compatibility to traditional food manufacturers with curative properties [33]. 

In arid areas such as the Middle East, green vegetation such as mangrove forest is scarce, and regions such as this require severe specific attention from the community and the government [34]. The satellite data show that mangrove forests in Saudi Arabia, Qatar, and Bahrain remain stable with a slight increase [35], while an intense incline is observed in the United Arab Emirates. The plantation of mangroves has driven the increase in the mangrove coverage area during the past decade [36]. The investigation of attractive mangrove forest products such as the fruits will encourage the rehabilitation program in the region.

Thus, this study summarizes the nutrition content along with secondary metabolites from mangroves and the possibility of the fruit being upgraded into functional food. The review is narrated in several parts, beginning with the nutrition content of the mangrove fruit followed by the secondary metabolites. In addition, the exploration of the metabolites is also extended to seed and fungus symbionts in the fruit. The processing of the mangrove fruit is later described as conveying the intermediate and final process. The current progress of exploration of mangrove plants in the Middle East is also defined.

## 2. Nutrition Composition and Bioactivity of Mangrove Fruit Extract

**Figure 1:**
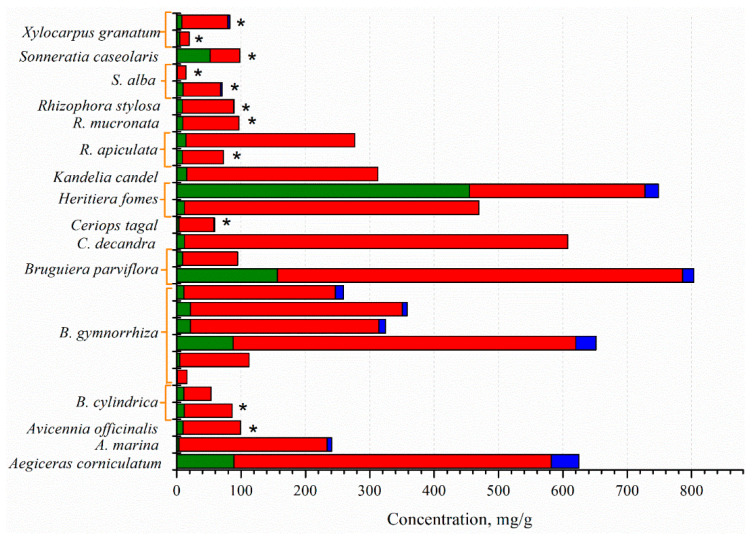
The primary metabolites from some selected mangrove fruit: 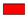, carbohydrate; 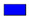, lipid; 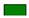, protein; *, carbohydrate is presented by total sugar and lipid is presented by fat. Modified from [41,42,43].

**Figure 2:**
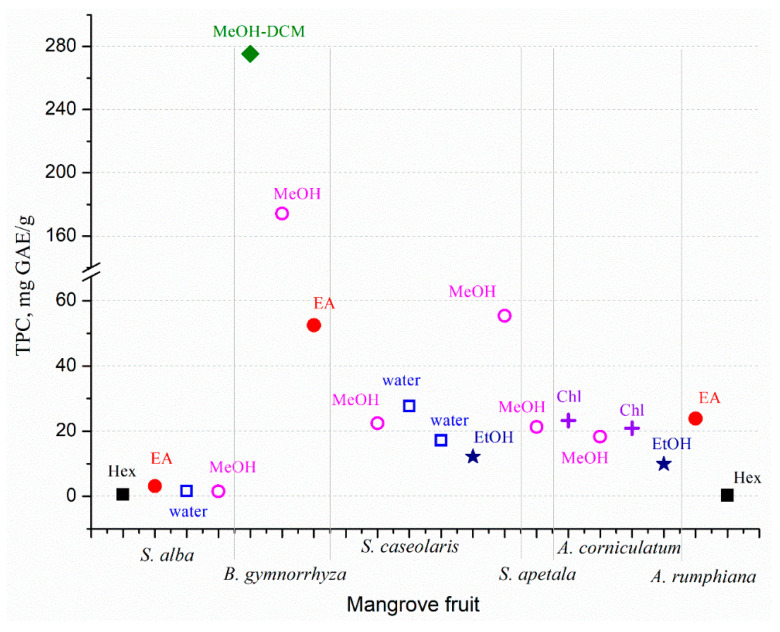
Total phenolic content (TPC) from several mangrove fruit extracted using different solvents: Hex (hexane), EA (ethyl acetate), water, MeOH (methanol), DCM (dichloromethane), EtOH (ethanol), and Chl (chloroform). Modified from [37,38,39,40,53,54].

The mangrove fruit contains diverse nutrition compositions concerning the species. Carbohydrate is the dominant nutrition in all mangrove fruit, while the protein and fat contents are varied (Figure 1). The total phenolic content (TPC) in the mangrove fruit is also widely investigated and, for most purposes, relates TPC with antioxidant activities [37,38]. TPC is positively correlated with the antioxidants; thus, the exploration probes for high TPC content in the fruit [39,40]. However, the evaluation is limited due to the extraction method (Figure 2), which may produce bias in the results. The TPC and other nutrient contents may be distinct among the fruit in the same species. Besides antioxidant activities, the mangrove fruit extract demonstrates other excellent bioactivities. 

The molecular study of the fruit extract of *A. officinalis* revealed the potentiality of the extract in treating the worldwide emerging coronavirus SARS-CoV-2. Four compounds within the extract—methyl palminoleate, methyl linoleate, hexacosane, and triacontane—showed excellent binding affinity to the virus’ main proteases, such as Arg188, Cys145, Gln189, Glu166, and Met165 [44]. However, long clinical steps are still required to produce firm outcomes in administering the mangrove fruit extract to treat SARS-CoV-2. 

The antibacterial activity of the mangrove extract comes from the synergistic relationship among secondary metabolites. The extract is rich in steroids, phenolic compounds, alkaloids, flavonoids, and other secondary metabolites. The enrichment of *Artemia salina* by extract of *S. alba* enhanced the resistance of giant tiger prawn against *Vibrio harveyi.* The enrichment of extract into *A. salina* escalates the steroid and phenol hydroquinone levels [45]. Alkaloid disrupts peptidoglycan in bacterial cell walls, leading to the death of cells, while phenol affects the cytoplasmic membrane and breaks the cell nucleus. Flavonoids modify the cell protein and DNA, resulting in the inhibition of the growth of the cell [46].

Extract of mangrove fruit exhibited antimicrobial activities (Table 1) due to many phytochemicals, such as the total phenolic content, flavonoid [47], saponin, tannin, alkaloids, and saponin [48]. The composition of those phytochemicals varies based on the extraction method and solvent used. The composition determines the bioactivity; therefore, selecting extraction procedures is crucial in achieving noticeable positive results. The common antibacterial mechanism is attributed to membrane cell disruption [49]. Despite the antibacterial activity, the extract also exhibits antiviral properties. The aqueous extract of *B. gymnorrhiza* demonstrates potent inhibition of Zika virus (ZIKV) infection on human epithelial A549 cells by preventing the binding of the virus to the host cell surface. The aqueous extract contains polyphenols that disrupt the lipid membrane (outer membrane) of the flavivirus [50]. The protein for the cell-binding receptor of ZIKV is targeted by cryptochlorogenic acid from the extract, leading to the virus’ death [51]. Moreover, the aqueous extract of *R. mangle* showed activity against various bacteria and depicted the cytotoxicity against human fibrosarcoma cell line HT1080 [52]. 

Various notable compounds are successfully identified from the extract of mangrove fruit. The eminent compound, such as (-)-17*β*-neriifolin, a cardiac glycoside, is found in the extract of *Cerbera manghas* with excellent heart stimulation function, effective in curing acute heart failure, at the same time show antiestrogenic, antiproliferative, and anticancer activities [55]. The fruit of *B. gymnorrhiza* contains isopimaradiene and 4-(2-aminopropyl) phenol. Isopimeradiene acts as antioxidative, anti-inflammatory, and antibacterial while 4-(2-aminopropyl) phenol shows high ROS scavenging activity, O_2_ scavenger, NF-E2-related factor 2 (Nrf2) stimulant, down-regulated cyclooxygenase-2 expression, and lowering of the nitrite level [38].

The administration of the mangrove fruit extract to animal models presents multiple vantages such as antioxidant [56], anti-atherosclerosis [57], antimicrobial [47,58], anti-diabetes [59], and hepatoprotective properties [60]. Excessive free-radical levels of diabetes trigger other diseases due to oxidative stress. Synthetic antioxidants are usually administered to reduce the oxidant level in the human body; however, side effects such as carcinogenicity should be addressed [61]. Mangrove fruit is a source of bioactive compounds with antioxidant activities. The antioxidant activity of the fruit is commonly related to the high content of vitamin C, anthocyanins, flavonoids, and polyphenols, which have hydrogen donating capabilities against free radicals such as nitric oxide (NO) [56]. The administration of methanol extract of the fruit of *S. apetala* to Long–Evans male rats inhibits the nitrite production in a dose-dependent manner. The extract acts as an insulin-like compound and modifies glucose utilization, enhances the transport of blood glucose to peripheral tissue, and stimulates the regeneration of the pancreas’ cells [40].

**Table 1:** The antimicrobial activities of fruit extract from different types of mangrove species.

Species	Solvent	Antimicrobial	Ref
*Avicennia marina*	Ethanol	*Aspergillus fumigatus*	[58]
		*Candida albicans*	
*A. officinalis*	Methanol	*Escherichia coli*	[47]
		*Enterobacter aerogenes*	
		*Klebsiella pneumoniae*	
		*Pseudomonas aeruginosa*	
		*Bacillus subtilis*	
		*Lactobacillus delbrueckii*	
		*Staphylococcus aureus*	
		*Streptococcus pyogenes*	
*B. gymnorrhiza*	Methanol	*E. coli*	[51]
		*P. aeruginosa*	
		*K. pneumoniae*	
		*S. aureus*	
		*Salmonella enteritidis*	
		*Sarcina lutea*	
		*Proteus mirabilis*	
		*Bacillus cereus*	
		*C. albicans*	
*R. mangle*	Ethanol	*Enterococcus faecalis*	[52]
		*Bacillus thuringiensis*	
		*Bacillus cereus*	
		*Streptococcus lactis*	
		*S. aureus*	
*S. apetala*	Methanol	*E. coli*	[40]
		*E. faecalis*	
		*Pseudomonas* sp.	
		*Shigella flexneri*	
		*Staphylococcus epidermidis*	
*S. caseolaris*	Ethyl acetate	*E. coli*	[48]
		*C. albicans*	
	Ethanol	*E. coli*	
		*S. aureus*	
		*C. albicans*	
	Methanol	*S. aureus*	[49,62]
		*E. coli*	
		*C. albicans*	
		*P. aeruginosa*	
		*Acenobacter baumannii*	
	Methanol:ethanol	*E. coli*	[63]
		*Klebsiella* sp.	
		*Shigella boydii*	
		*S. sonnei*	
		*S. aureus*	
*X. mekongensis*	Methanol:ethanol	*S. aureus*	[63]

The ethanolic extract of *S. apetala* shows anti-atherosclerosis in the male Wistar rat model [57]. The cholesterol levels, including LDL and HDL, are higher, and the formed foam is lower than the control animal [57]. Atherosclerosis is a vascular inflammatory disease characterized by lipid accumulation, fibrosis, and cell death in the arteries. The inflammation is mainly caused by high free-radical concentration ensuing in the incline of plasma lipid levels, such as LDL. LDL infiltrates the vascular sub-endothelium via impaired endothelium, which excites the oxidation process [64]. The oxidized LDL activates the endothelial cells expressed by leukocyte adhesion molecules such as vascular cell-adhesion molecule 1 (VCAM-1) on the surface of the artery. The elevated oxidized LDL stimulates the production of ROS via nitric oxide activation. Typically, NO is a protective substance produced by the vascular endothelial cells; however, it enables pro-atherogenesis if produced by macrophages. Moreover, excessive ROS generation stimulates and activates Nf-kB p65 translocation, increasing the expression of monocyte chemoattractant protein (MCP-1) and releasing the pro-inflammatory mediators by macrophages. Monocyte turns phagocytosis and macrophages of oxidized LDL to form foam cells [65,66,67]. Regarding its antioxidant property, the mangrove fruit extract prevents the excessive production of free radicals at the beginning of the mechanism [64].

The administration of the ethyl acetate extract of mangrove fruit *X. moluccensi* presented antidiabetic properties in a male albino Sprague–Dawley rat model; many blood parameters such as the blood glucose, serum fructosamine, serum triglycerides, and serum cholesterol levels declined. The extract improves phosphofructokinase and pyruvate kinase in the liver and kidneys while maintaining bodyweight [59]. The fruit of *S. apetala* displays hepatoprotective properties of male Kunming mice. The fruit extract’s antioxidative property improves the aspartate aminotransferase level in serum, reduces the alanine aminotransferase, and increases the survival rate. The hepatoprotective ability of the fruit extract in the liver is depicted by the incline in total antioxidant capacity and catalase, improvement in glutathione peroxidase and glutathione, and inhibition of myeloperoxidase, interleukin 6, and tumor necrosis factor-α. The antioxidant properties of the fruit extract are suspected to prevent the liver damage caused by oxidative stress from ROS [60]. 

## 3. Secondary Metabolites Isolated from Mangrove Fruit

The exploration and discovery of secondary metabolites in mangrove fruit are highly valued, mainly for pharmaceutical and nutraceutical purposes. The intensive investigation is organized to isolate the potent secondary metabolites. Protolimonoids and limonoids are common secondary metabolites found in mangrove fruit. Both compounds are triterpenoid with four rings and a *β*-substituted furanyl ring at the 17*α* position. The differences are observed in the sidechain and composition of the ring. The sidechain and all rings of the protolimonoids are intact; meanwhile, the sidechain of limonoid is furan with the substitution of lactone at ring D. Limonoids such as mexicanolide and phragmalin possess an opened and recycled skeleton in ring B, whilst phragmalin is indicated by specific C4, C29, and C1 bridges. Mexicanolides are a precursor for phragmalins. All limonoids are in the oxygen-containing group at C-3, C-4, C-7, C-16, and C-17 [68].

**Figure 3:**
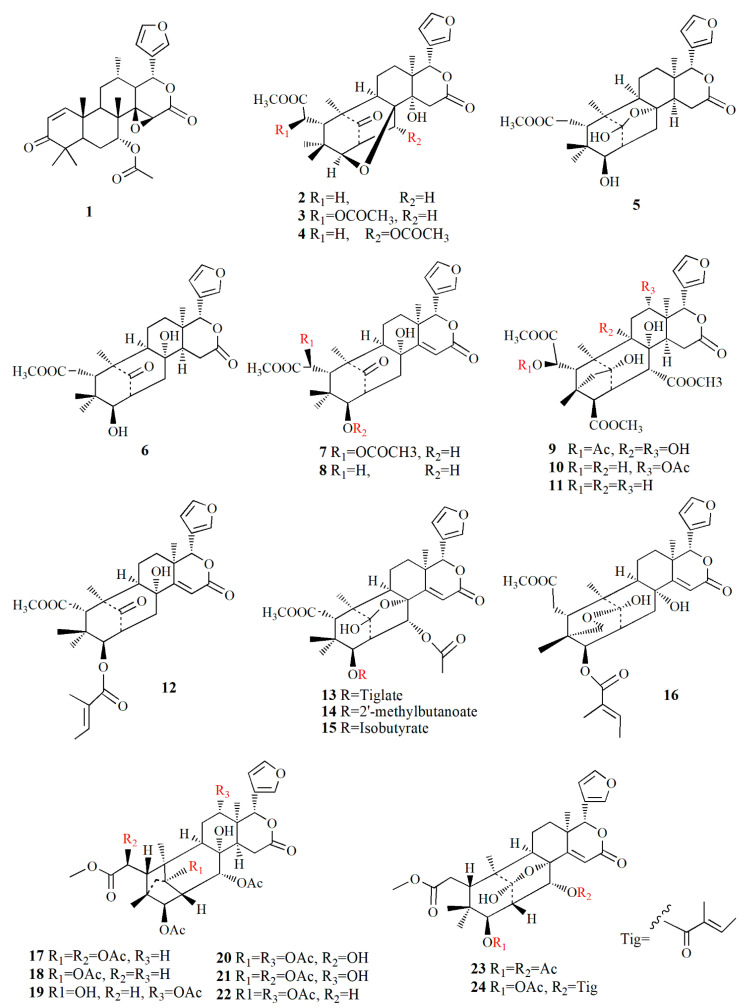
The molecular structure of compounds **1**–**24**.

**Figure 4:**
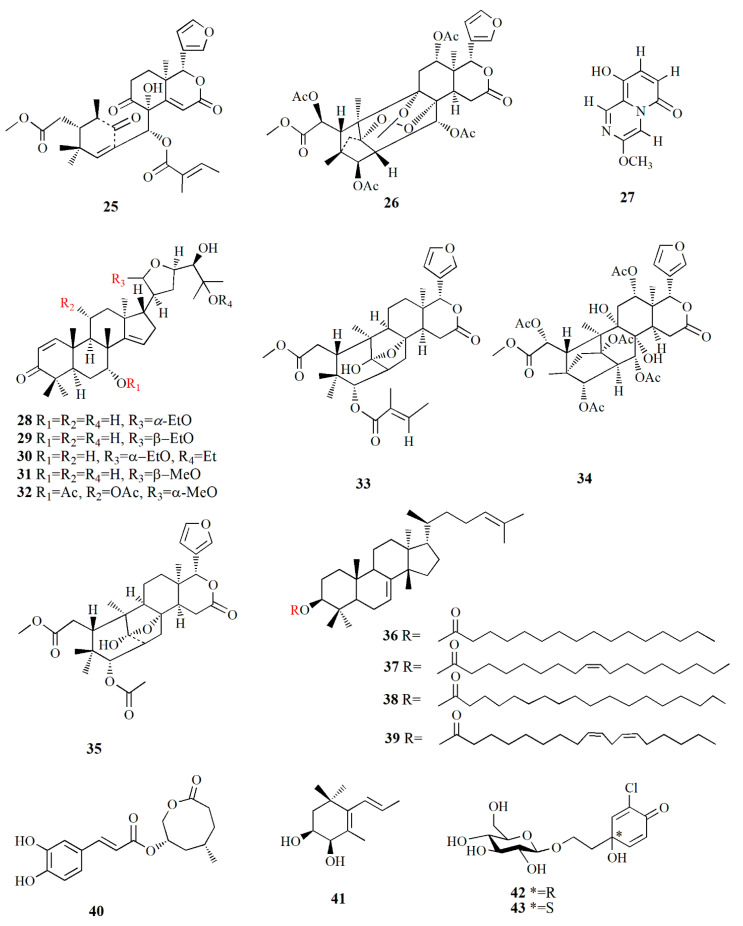
Molecular structure of compounds **25**–**43**.

**Figure 5:**
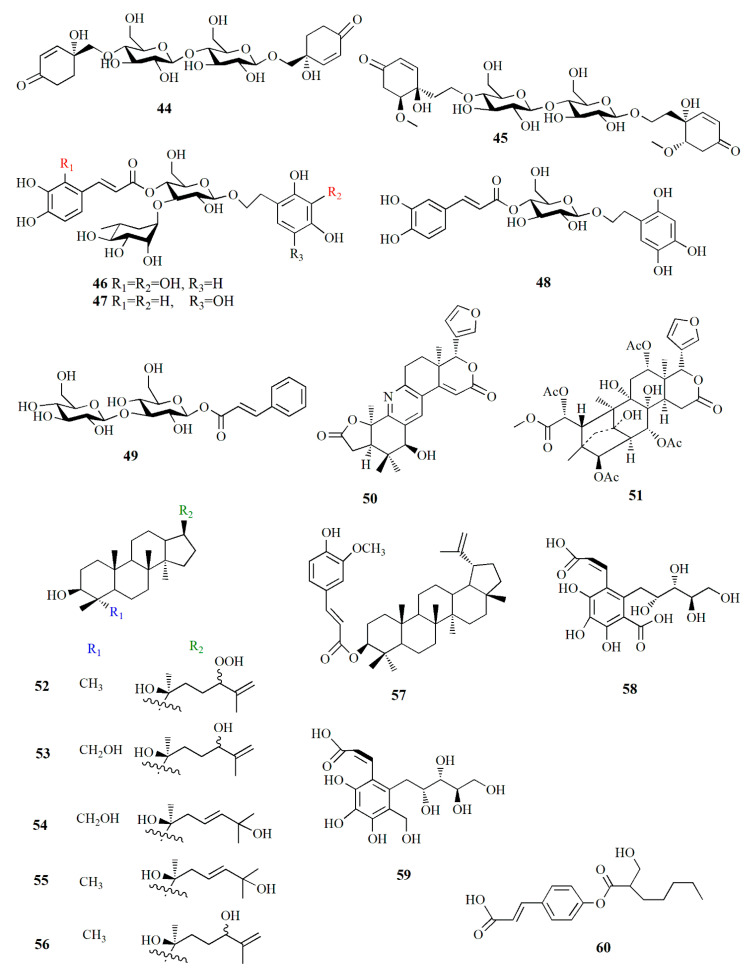
Molecular structure of compounds **44**–**60**.

**Figure 6:**
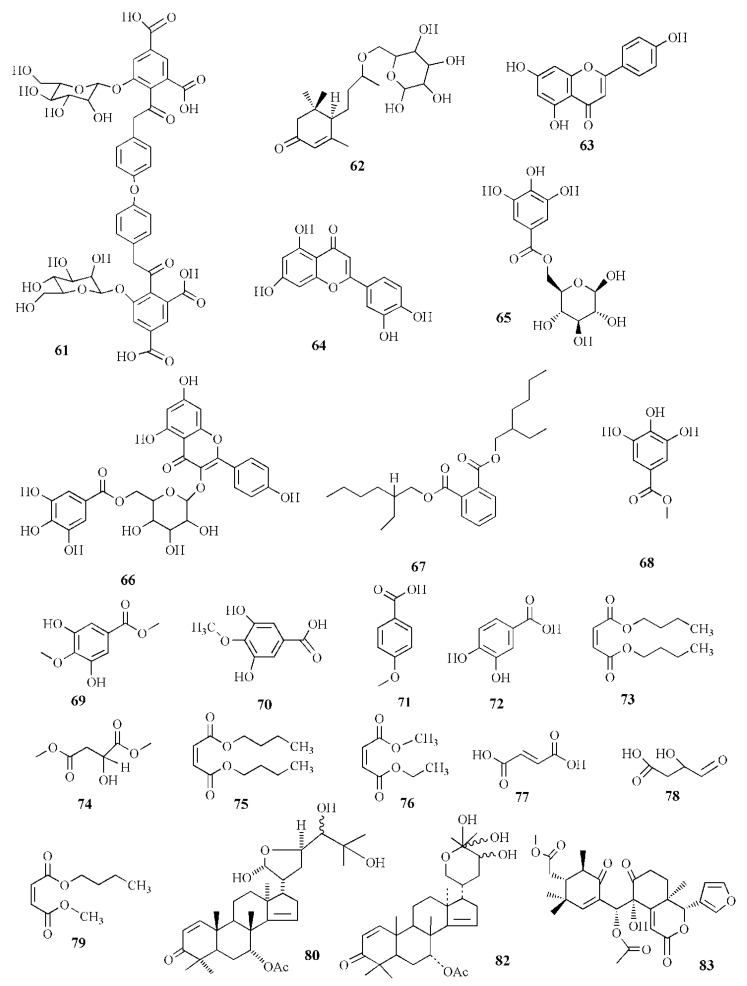
Molecular structure of compounds **61**–**83**.

**Figure 7:**
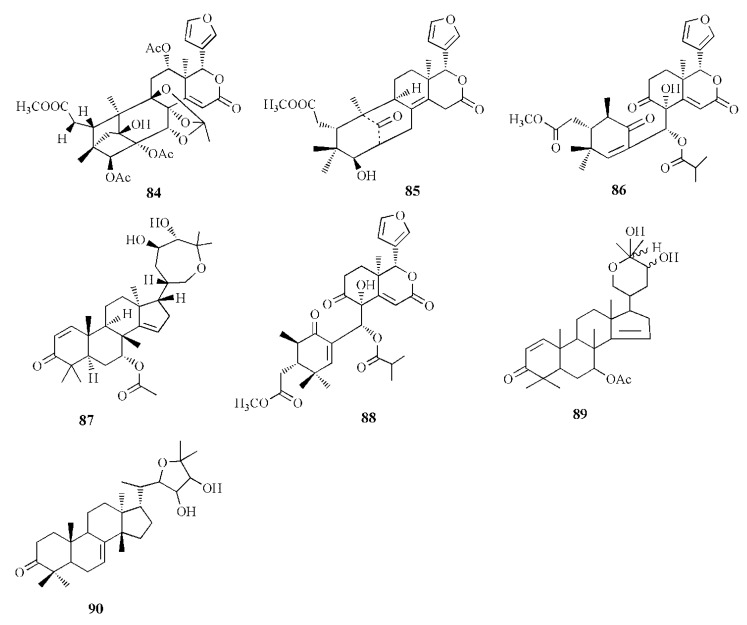
Molecular structure of compounds **84**–**90**.

The genera *Xylocarpus*, *Avicennia*, and *Sonneratia* are commonly explored for their fruit phytochemistry. The fruit of those genera are famous for the biosynthesis of limonoids (Table 2) with similarities in their molecular structures (Figure 3, Figure 4, Figure 5, Figure 6 and Figure 7). *X. granatum* is used in traditional medicine in southeast Asia to treat antifeedant, malaria, chlorella, diarrhea, and other diseases. Limonoids and derivatives such as polyoxyphragmalin and mexicanolide compounds are common isolates from *X. granatum*. The fruit of the mangrove is rich in polyoxyphragmalins; meanwhile, mexicanolides can be found either in seed or fruit [69]. Gedunin (**1**) isolated from the fruit of *X. granatum* inhibited the pharyngeal cancer FaDu, colon cancer DLD-1, lung cancer A549, ovarian cancer OVCAR-3, and PA-1. In ovarian cancer cells, the administration of **1** showed G2/M cell-cycle arrest, leading to cell death in a dose- and time-dependent manner. The death of cancer cells is promoted by disturbance in the regulation of cell-cycle protein, provoking nucleus fragmentation, pyknosis, and cell shrinkage. Compound **1** induced apoptosis by stimulating mitochondria to release cytochrome c, followed by proteolytic cleavage of many key proteins, including poly (ADP) ribose polymerase (RARP) [70].

Xyloccensin-type limonoids are isolated from various fruits of the genus *Xylocarpus*. The ethanol extract of *X. granatum* collected from Hainan, China, afforded 3*β*,8*β*-epoxymexicanolide, a rare oxidized limonoid. Xyloccensin K (**2**) has a 3,8-epoxy bridge, which is shown by the relationship between H-3 at δ_H_ 4.13 ppm and C-8 at δ_C_ 81.37 ppm. The α-configuration was depicted by the correlation between H-30 at δ_H_ 5.52 ppm, H-2 at δ_H_ 3.09 ppm, and H-3 and H-29 at δ_H_ 0.57 ppm. The extract also contains 6-acetoxycedrodorin (**3**) and xyloccensin W (**4**) (C_29_H_36_O_10_); **4** possesses *β*-furyl ring, acetyl, a methoxy, a hydroxyl, and a ketone function. Compounds **2**–**4** are similar in their ring skeleton, where the difference is demonstrated by the arrangement of the acetyl group attached to C-6 and C-30 [71]. 

Ethanol extract of *X. granatum* fruit from Hainan Island contained 3-deacetyl xyloccensin M (**5**) with a molecular formula of C_27_H_36_O_8_. Compound **5** has six rings, two esters, and carbon–carbon double bonds, and it has a similar structure to xyloccensin M. Another isolated compound, 3-deacetyl xyloccensin N (**6**) with a molecular formula, C_27_H_36_O_8_, has keto and ester carbonyl groups, hydroxyl, carbon double bond, one methoxycarbonyl group, and *β*-furyl moiety [87]. Compounds **5** and **6** are a pair of isomers of mexicanolides, and compound **6** is suspected as a biosynthetic intermediate of compound **5** [72]. These two compounds resembled mexicanolides isolated from the stem bark of *X. granatum*, namely xyloccensin M and xyloccensin N with the absence of the acetyl group [88]. Within *X. granatum*, xyloccensin X_1_ (**7**), C_29_H_36_O_10_, was observed with methoxycarbonyl at δ_H_ 3.74 ppm and δ_C_ at 53.5 and 172.6 ppm while the *β*-furyl ring at δ_H_ 6.57, 6.53, and 7.68 ppm related to carbons at δ_C_ 111.6, 121.6, 143.7, and 144.4 ppm. The extract also yielded xyloccensins X_2_ (**8**) with a molecular formula of C_27_H_34_O_8_. Compound **8** is similar to compound **7** without the 6-acetoxy group [73]. 

Ethanolic extract of *X. granatum* from Hainan, China contains xyloccensin Y **(9)** and xyloccensin Z1–Z2 (**10**–**11**). Compound **9**, C_33_H_42_O_15_, is a hexacyclic with a *β*-furyl ring; three acetyls; a methoxy, methoxycarbonyl; and three methyls of the phragmalin nucleus. Compound **10** has a molecular formula of C_33_H_42_O_14_, and the backbone of its structure is similar to compound **9** without the hydroxyl group. Compound **11**, C_31_H_40_O_12_, is similar to that of compound **10**, except for the absence of the 12-acetoxy group. Phragmalins with an 8,9 hydroxy group are less common in the maliaseae plants. Limonoids have intact oxidized triterpene nuclei, and they are triterpene derivatives from a precursor 4,4,8-trimethyl-17-furanylsteroid backbone [74].

The ethanolic extract of *X. granatum* fruit confines xylogranatin A–D **(12–15).** Compound **12**, C_32_H_40_O_9_, is mexicanolide with a *β*-furyl ring, tiglate group, a methoxycarbonyl group, a ketone, and an α,*β-* unsaturated δ-lactone ring D. Compound **13** (C_34_H_42_O_11_) is similar to **12** with the addition of an acetoxy group at δ_H_ 1.9 ppm and δ_C_ at 20.9, 170.2 ppm, and acetal carbon at δ_C_ 107.4 ppm with the absence of ketone carbon at δ_C_ 218.2 ppm. Compound **14** (C_34_H_44_O_11_) resembles **13** with the absence of the tiglate group and replaced by 2’-methyl-butanoate. Compound **15**, C_33_H_42_O_11_, resembles compound **13** with the absence of a tiglate group and replaced by an isobutyrate group [75]. A phragmalin is isolated from ethanol extract of fruit *X. granatum*, namely xylogranatin E (**16**), C_32_H_40_O_10_, and it has a rare oxygen bridge of C1 to C29. The compound is characterized by six unsaturated units represented by six rings, *β*-furyl ring, a tiglate moiety, a methoxycarbonyl, and an *α,β*-unsaturated δ-lactone ring E [76]. 

Ethanol extraction of *X. granatum* fruit led to the isolation of several limonoids, i.e., xylocarpin A–I (**17**–**21, 23**–**26**) and 6-dehydroxyxylocarpin (**22**). Compound **17**, C_35_H_44_O_14_, is characterized by a *β*-substituted furan ring at δ_H_ 7.45, 6.38, and 7.44 ppm, four oxymethines, seven methyls, and four acetyl groups. Compound **18** (C_33_H_42_O_12_) shares the same basic skeleton as **17**, with the presence of a methylene group at δ_H_ 2.25 and 2.31 ppm, while it loses the C-6 oxymethine signal. Compound **19**, C_33_H_42_O_13_, is similar to compound **17** with the shift of the C-1 signal to δ_C_ 81.5 ppm. Compound **20** is similar to compound **19**, except for the presence of seven oxygenated carbons at δ_C_ 69.8, 77.3, 71.3, 73.4, 71.2, 76.4, and 88.0 ppm. Compound **21** is similar to compound **20** with the oxygenated carbons at C-1, C-3, C-6, C-8, C-12, and C-30; meanwhile, the significant downfield of H-6 was observed at δ_H_ 5.40 ppm. Compound **22**, C_35_H_44_O_14_, is a phragmalin with a *β*-substituted furanyl group. Compound **23**, C_31_H_38_O_11_, contains four tertiary methyl groups, two acetyl methyl groups, a methoxy group, and two oxymethine protons. Compound **23** possesses the same molecular formula as xylogranatin B with an acetoxy group at C-3 and tigloxy group at C-30. Compound **24**, C_32_H_38_O_10_, is composed of three tertiary methyls and a *β*-substituted furan ring. Compound **25**, C_37_H_44_O_16_, is uniquely characterized by 1,1,1-trioxyethyl moiety, as represented by the relationship between methyl singlet at δ_H_ 1.66 ppm and quaternary carbon at δ_C_ 119.2 ppm [69]. Ethanolic extract from *X. granatum* from Hainan, China, afforded pyrazine alkaloid xylogranatinin (**27**), C_9_H_8_N_2_O_3_. It is a bicyclic molecule with a pyridine ring that is configured as *α,β*-unsaturated lactam [77]. 

Ethanol extract of *X. granatum* fruit contains five protolimonoids, 2 limonoids, and 2 xylocarpins. Protoxylocarpin A (**28**), C_32_H_50_O_6_, is characterized by the presence of C=O and OH groups. Compound **28** shows characteristics of apotirucallane triterpene with the tetracyclic nucleus. Protoxylocarpin B (**29**) is similar to compound **28**, except for the occurrence of the C17 shifting to δ_C_ 52.4 ppm and C21 to δ_C_ 103.3 ppm. The structure of Protoxylocarpin C (**30**), C_34_H_54_O_6_, resembles compound **28**, with the addition of ether groups appearing at δ_C_ 56.5 ppm and 16.1 ppm. Ether groups are resumed from the link of the ether with HO-C25 moiety at δ_C_ 76.6 ppm. Protoxylocarpin D (**31**) is identical to holstinone B, with a difference being observed in the carbon shift of C21 at δ_C_ 104.6 ppm and C17 at δ_C_ 52.5 ppm. Protoxylocarpin E (**32**), C_35_H_52_O_9_, is an apotirucalla-1,14-dien-3-one analogue. 

Xylocarpin J (**33**), C_32_H_42_O_9_, is a C3 epimer of xylocarpin A. Xylocarpin K (**34**), C_37_H_46_O_17_, is phragmalin limonoid and configured by five AcO groups. Xyloccensin M (**35**) is also isolated from the same extract. Compounds **28**–**33** and **35** showed weak-to-moderate activity against HCT-8, Bel-7402, BGC-823, and A2780 cell lines [78]. 

Protolimonoids and their fatty acid are isolated from the ethanol extract of *X. granatum*. Alkaline hydrolysis of the ethanol extract using sodium hydroxide produces two mixtures: a mixture of fatty acids, and a mixture of butyrospermol and *β*-sitosterol. The protolimonoids are identified as butyrospermols, i.e., butyrospermol 3*β-O*-palmitate, C_46_H_80_O_2_ (**36**), butyrospermol 3*β-O*-oleate, C_48_H_82_O_2_ (**37**), butyrospermol 3*β-O*-stearate, C_48_H_84_O_2_ (**38**), and butyrospermol 3*β-O*-linoleate C_48_H_80_O_2_ (**39**). The composition of the fatty acid mixture is 3.3% methyl myristate, 62.2% methyl palmitate, 20.1% methyl oleate, 9.8% methyl stearate, and 4.5% methyl linoleate [79]. Ethanolic extract of *A. marina* fruit from Guangxi province, China, contains caffeoyl derivatives such as maricaffeolylide A, C_17_H_20_O_6_; (**40**) and maricyclohexene A, C_12_H_20_O_2_ (**41**). The caffeoyl moiety of compound **40** is presented by proton at δ_H_ 7.02, 6.93, and 6.77 ppm, and it possesses an eight-membered lactone ring with two chiral centers. Compound **41** confirms its cyclohexene group as a positive cotton effect by CD spectrum at 205 nm. Compound **40** exhibited cellular antioxidant assay (CAA) with an EC_50_ value of 24±0.3 μM, and compound **41** showed an EC_50_ value of 339 ± 3 μM [80]. 

Jacaranone analogs marinoid F–I (**42**–**45**) are isolated from *A. marina* fruit from Beibu Gulf, China, using an ethanol–dichloromethane solution. Compound **42**, C_14_H_19_ClO_8_, contains an *α,β*-unsaturated carbonyl group resonating at δ_H_ 6.22, 6.99, and 7.12 ppm; and two methylene groups at δ_H_ 2.08, 3.64, and 3.94 ppm. The compound is distinguished by its *β*-glucosyl group and para-quinol-type partial structure. Compound **43**, C_14_H_19_ClO_8_, is similar to compound **42** except for the cotton effect (∆ε) of **42,** which is –4.28 while that of compound **43** is +5.97. Compound **44**, C_26_H_38_O_15_, possesses two cyclohexanone moieties with two *β*-glucosyl groups. Compound **45**, C_30_H_46_O_17_, contains two cyclohexanone moieties and is considered an unsymmetrical dimer of cornoside analog. Compound **45** exhibited the strongest cellular antioxidant assay with an EC_50_ value of 26 μM, while compounds **42**–**44** were weak with EC_50_ values of 598, 4971, and 1103 μM, respectively [81].

Phenylethyl glycosides and cinnamoyl glycosides, named marinoid J–M (**46**–**49**), are isolated from the ethanol–dichloromethane extract of *A. marina* fruit collected from Beibu Gulf, China. Compound **46**, C_29_H_36_O_17_, possesses 2,3,4-trihydroxyphenyl moiety and 2,3,4-trihydroxycinnamoy moiety in the AB system. Compound **47**, C_29_H_36_O_16_, contains 2,4,5-trihydroxyphenyl moiety, two trans-olefinic protons in the AB-type system, and caffeoyl moiety. Compound **48**, C_23_H_26_O_12_, presents *trans*-olefinic protons in AB-type, caffeoyl moiety, 2,4,5-trihydroxyphenyl moiety, and *β*-glucosyl. Compound **49**, C_21_H_28_O_12_, is composed of two *trans*-olefinic protons in AB-type, phenyl moiety, two *β*-glucosyls, and cinnamoyl moiety. Cellular antioxidant assay of compounds **44**–**49** showed EC_50_ values of 23, 36.2, 114.5, and 247.8 μM, respectively [82].

A limonoid-based alkaloid granatione (**50**), C_26_H_27_NO_6_, is isolated from ethanol extract of *X. granatum* fruit, which possesses conjugated δ-lactones in rings E and B substituted furan ring. The cyclohexene D of the compound fused to the δ-lactone ring. Compound **50** has five aromatic signals on the carbon at δ_C_ 158.2, 124.3, 133.9, 130.5, and 156.6 ppm assigned as tetra-substituted pyridine units. The *γ*-lactone is formed by carbonyl carbon and a methylene group. Compound **50** was biosynthetically obtained from 9,10-seco-mexicanolide xylogranatin C as a precursor. A phragmalin xylocarpin L (**51**), C_35_H_44_O_16_, is isolated from the same extract [83].

Dammarane triterpenes are isolated from the hexane and dichloromethane extract of *C. tagal* collected at Nakhon Si Thammarat, Thailand. Cereotagaloperoxide (**52**), C_30_H_52_O_4_Na, is characterized by a tetracyclic dammarane with five methyl singlets and oxymethine proton**.** Cereotagalol A (**53**), C_30_H_52_O_4_, is similar to compound **52** with the addition of one hydroxymethylene group and the loss of a methyl group. Cereotagalol B (**54**) is similar to compound **53** in its tetracyclic moiety, and the difference was observed as two olefinic methine protons instead of terminal methylene protons. Isofouquierol (**55**), fouquierol (**56**), and 3*β-E*-feruloylbetulinic acid (**57**) are isolated from the same extract. Compound **57** showed cytotoxic activity against human breast cancer KB, human oral epidermoid carcinoma BC, and human small-cell lung cancer NIC-H187 cell line with IC_50_ values of 3.8, 3.0, and 1.8 μg/mL, respectively [84].

Alcoholic extract of *S. apetala* fruit produced types of sonneradons. Sonneradon A **(58)**, C_15_H_18_O_11_, has an *α,β*-unsaturated carbonyl group from proton δ_H_ 8.07 and 7.52 ppm and carbon at δ_C_ 113.9, 149.1, and 172.8 ppm. Sonneradon B (**59**), C_15_H_20_O_10_, also possesses an *α,β*-unsaturated carbonyl group at proton δ_H_ 8.16; 7.41 ppm along with *ortho*-trihydroxyphenyl moiety and 2-deoxy-D-arabino-hexitol moiety. Sonneradon C (**60**), C_17_H_22_O_5_, contains two AB system proton signals at δ_H_ 6.80 and 7.58 ppm as 1,4-disubstituted phenyl moiety, and Sonneradon D (**61**) has the molecular formula C_44_H_42_O_23_. Other compounds have been identified, and these compounds are isolated from other types of plants, viz. ranuncoside (**62**), apigenin (**63**), luteoline (**64**), 6-*O*-galloyl-*d*-glucopyranose (**65**), *O*-*B*-(6-*O*-galloyl)-glucopyranoside (**66**), 2-ethylhexyl phthalate (**67**), methyl gallate (**68**), methyl 4-*O*-methylgallate (**69**), 4-*O*-methylgallic acid (**70**), 4-methoxybenzoic acid (**71**), 3,4-dihydrobenzoic acid (**72**), bibutyl malate (**73**), dimethyl malate (**74**), bibutyl malate (**75**), ethylmethyl malate (**76**), 2-butenedioic acid (**77**), 3-hydroxy-4-oxobutanoic acid (**78**), and butylmethyl malate (**79**). Compounds **58**, **59**, **61**, **63**, **74**, and **79** significantly expanded the life span of nematode *Caenorhabditis elegans* as compound **58** was the most potent in the molecular docking analysis. Compounds **58**, **59**, **61**, **63**, **74**, and **79** showed heat-stress properties due to the interaction between the key amino residues of HSF-1 such as GLN49, ASN74, and LYS80 of the DBD (*N*-terminal DNA-binding domain). Heat-shock transcription factor-1 (HSF-1) is used for modulating the life span of an organism, maintaining proteostasis, and the heat-shock response. Compound **58** significantly extended the lifespan of the *N*2 worm by 34.48±0.92% from 15.08 ± 0.74 days to 20.28 ± 0.84 days [85].

Acetonitrile extraction assisted with an ultrasonic water bath method was able to isolate some limonoids from the fruit of *X. granatum*. Piscidinol G (**80**), xylogranation D (**81**), spicatin (**82**), xylogranatin C (**83**), xyloccensin V (**84**), proceranolide (**85**), xylomexicanin D (**86**), Sapelin E acetate (**87**), xylomexicanin A (**88**), grandifoliolenone (**89**), and odoratone (**90**) are quantitatively detected using LC/MS [86].

## 4. The Secondary Metabolites from the Seed of Mangrove

**Figure 8:**
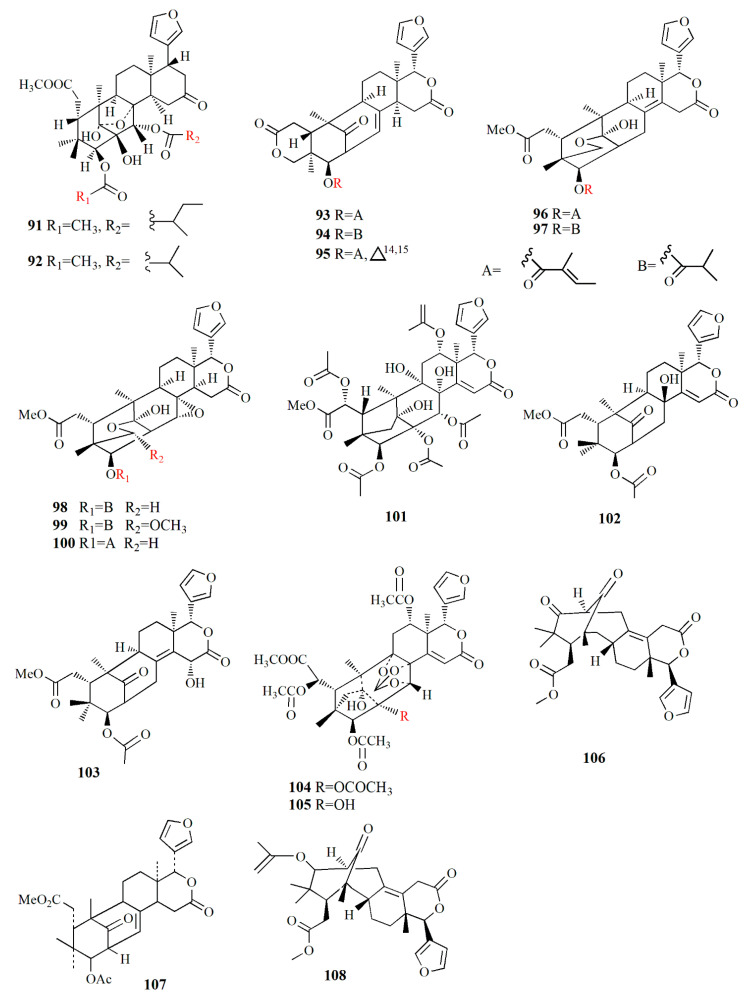
Molecular structure of compounds **91**–**108**.

**Figure 9:**
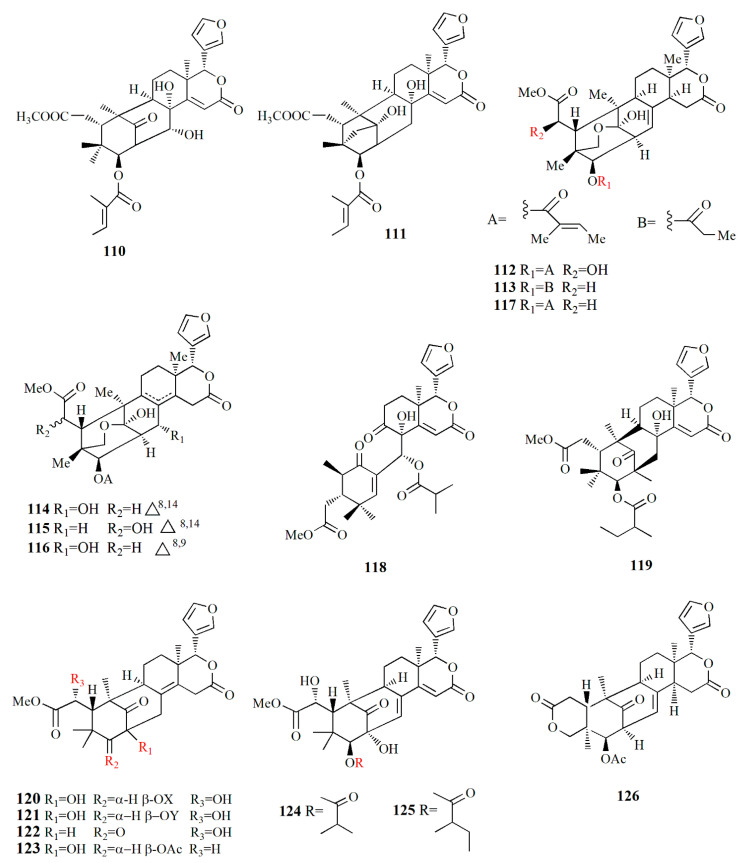
Molecular structure of compounds **110**–**126**.

**Figure 10:**
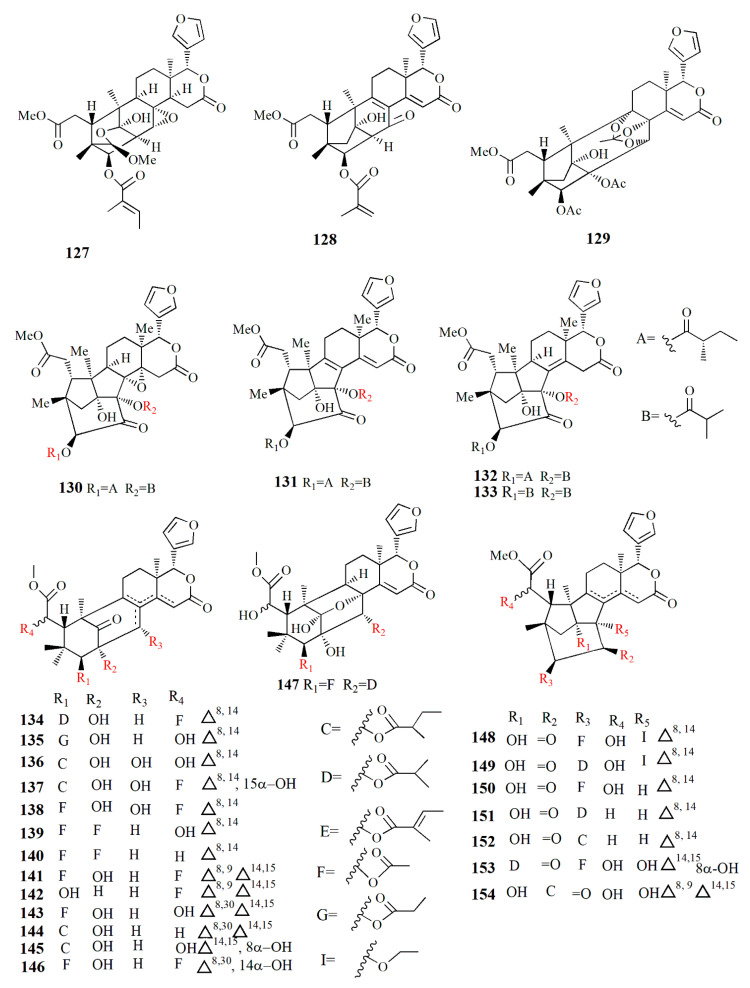
Molecular structure of compounds **127**–**154**.

**Figure 11:**
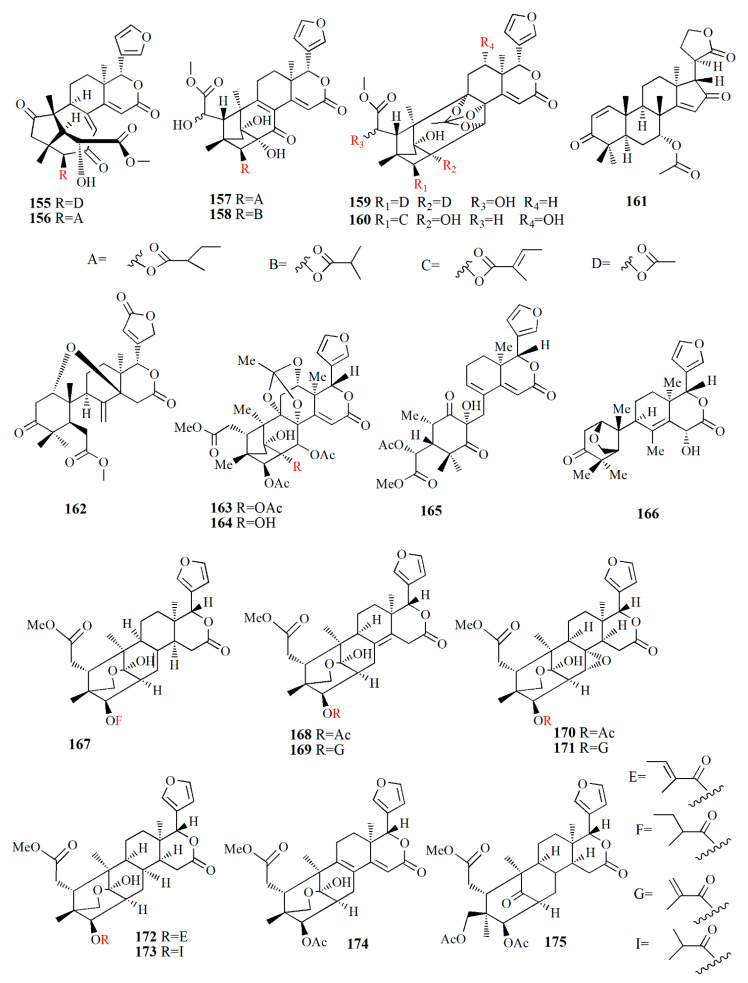
Molecular structure of compounds **155**–**175**.

**Figure 12:**
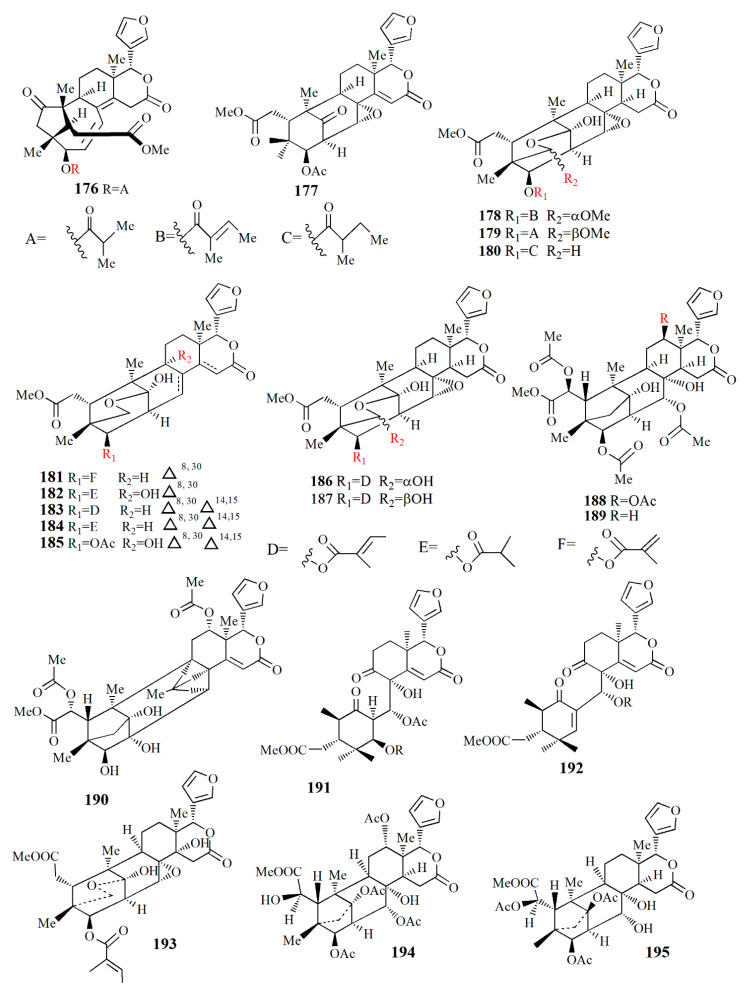
Molecular structure of compounds **176**–**195**.

**Figure 13:**
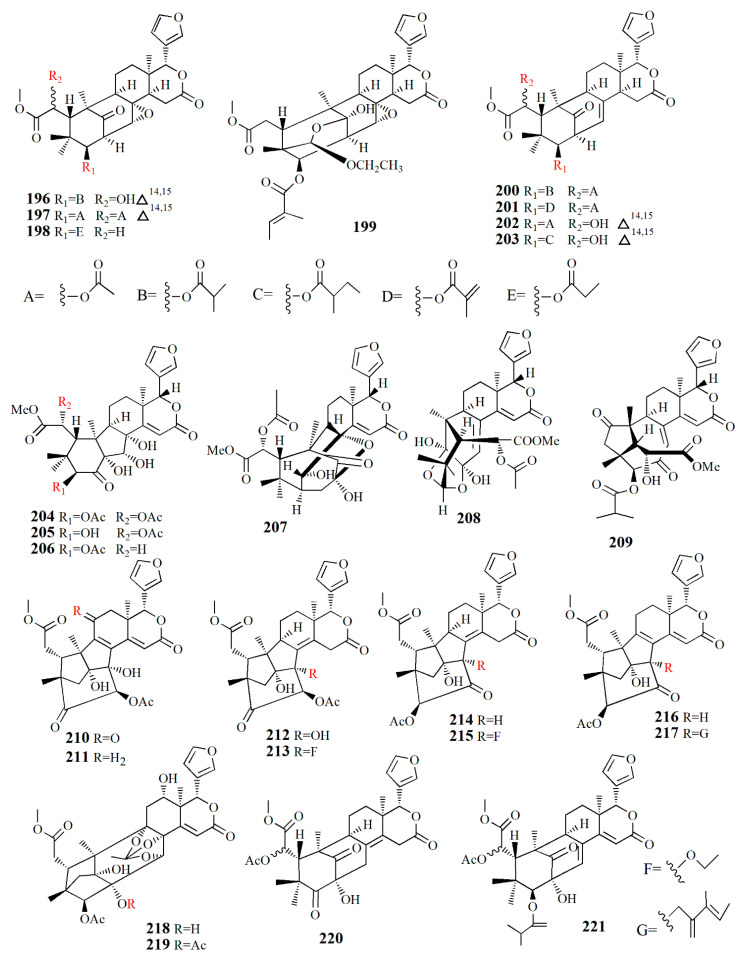
Molecular structure of compounds **196**–**221**.

**Figure 14:**
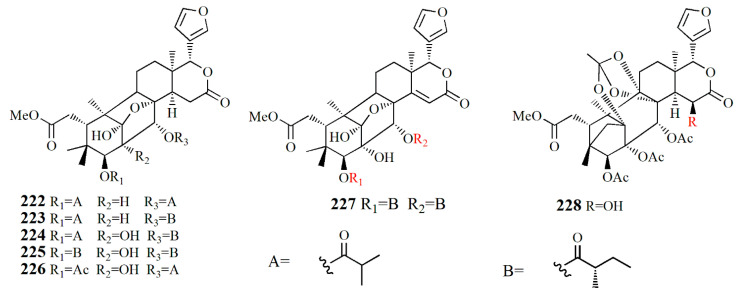
Molecular structure of compounds **222**–**228**.

The mangrove seed, the fruit’s flesh, and the pericarp are also consumed for their ethnomedicinal properties. The seed is rich in limonoid and its derivatives (Figure 8, Figure 9, Figure 10, Figure 11, Figure 12, Figure 13 and Figure 14) have various bioactivities (Table 3). The extract of *S. apetala* seed demonstrated anti-diarrheal and antimicrobial activities against *Staphylococcus aureus*, *Shigella dysenteriae*, *Salmonella* Typhi, *S. Paratyphi*, and *E. coli* [63]. 

Xyloccensin I (**91)**, C_34_H_46_O_12_, and Xyloccensin J (**92**), C_34_H_44_O_12_, were observed in the nonpolar extract of fruit kernel of *X. granatum* and *X. moluccensis*. Usually, limonoids are superior to insect antifeedants; however, these compounds show no antikinase, antiviral or antimicrobial activity [89]. Limonoids godavarins A–J (**93**–**99, 101**–**105**), xyloccensin L (**100**), xyloccensin P–Q (**104**–**105**), angustidienolide (**106**), 6-deoxy-3-detigloyl-swietenine acetate (**107**), fissinolide (**108**), and methyl 3*β*-acetoxy-1-oxomeliaca-8(9),14-dienoate (**109**) were observed in the seed of *X. moluccensis*. Compound **93** is characterized by the presence of ∆^8,30^ double bonds. Compound **94** resembles compound **93** by the addition of an isobutyryl group and loss of the 3-tigloyl group. Compound **94** is identical to compound **93**, except for the presence of a ∆^14,15^ double bond. Compound **95** is similar, with xylogranatinin E and the loss of a ∆^14,15^ double bond, while compound **96** is similar to compound **95** with the addition of the isobutyryl group. Compound **97** reflects xyloccensin L with the addition of the isobutyryl group, while compound **98** resembles that of compound **6** with the loss of the C-29 methylene group. Compound **99** is similar to granatumin G with the addition of two acetyl groups. Compound **100** is similar to xyloccensin X_2_ with the addition of the acetyl group. Compound **101** resembles 3-*O*-acetylswietenolide with an OH group located in C-15. Compounds **93**, **96**, **103**, and **106**–**109** exhibited insecticidal and antifeedant activities [90].

A mexicanolide, 30*α*-hydroxyl xylogranatin A (**110**), C_32_H_40_O_10_, and a phragmalin, xylogranatin E_2_ (**111**), C_32_H_40_O_9_, were isolated from ethanol extract of the seed of *X. granatum.* Both compounds have a 3-*O-β*-tigloyl group. Compound **110** is similar to xylogranatin A with the addition of a hydroxyl group attached to C30, while compound 111 is similar to xylogranatin E without an oxygen bridge between C-1 and C-29 [91]. Ethanol extract of the species also contains thaigranatins A–E (**112**–**116**) and granatumin L (**117**). Compounds **112**–**116** contain an oxygen bridge between C1 and C19. Compound **112** contains a typical *β*-furyl ring, oxygenated methylene moiety, tigloyl group, and methoxycarbonyl moiety. Compound **113** is similar to compound **112** with the presence of 3-*O*-propionyl moiety instead of a 3-*O*-tigloyl group. Compound **114** and compound **115** are similar to godavarin D with the addition of a 30-OH group for compound **114** and a 6-OH group for compound **115**. Compound **116** is similar to compound **114** with the presence of ∆^8,9^ double bonds instead of ∆^8,14^ double bonds. Compound **117** was active against HIV-1 with an inhibition rate of 67.10 ± 3.04 % at 20 μM. The derivatization of compound **117** is carried out by employing several protocols, i.e., oximization with hydroxylamine, esterification with diazomethane along with other organic compounds, and alkalization by KOH. However, among the derivatives, esterification by diazomethane of alkalinized compound **6** is the only active compound against HIV-1. The C3 substituent of the compound is the determining group for anti-HIV-1. Fatty acid ester in C3 is the best substituent compared to C=N or C=O [92].

∆^14,15^-mexicanolides were isolated from the ethanol extract of *X. granatum* seed, namely xylomexicanins A and B (**118**–**119**). Compound **118** contains an isobutytyloxy group, a methoxycarbonyl group, a hydroxyl group, and an *α,β*-unsaturated keto carbonyl group. Compound **119** resembles that of xylogranatin A, with the difference being in the replacement of tiglate moiety by 2’-methylbutanoyl moiety. Compound **118** exhibits antiproliferative activity against KT human tumor cell [93].

Moluccensins R–Y (**120**–**121, 124**–**129**), 6-hydroxymexicanolide (**122**), and 2-hydroxyfissinoide (**123**) were isolated from *X. moluccensis*. Compound **120** has isobutyryl moiety, and it resembles that of 2,6-dihydroxyfissinolide. Compound **121** is similar to **120,** with the presence of 3-*O*-2-methylbutyryl moiety instead of a 3-*O*-isobutyl group. Compound **122** was first discovered as an oxidation product of swietenolide. Compound **124** resembles that of **120**, with the addition of two conjugated double bonds: ∆^8,9^ and ∆^14,15^. Compounds **124** and **125** are structural analogues. Compound **126** is similar to godavarin A with a 3-*O*-acetyl moiety instead of the 3-*O*-tigloyl moiety. Compound **127** resembles that of godavarin G with the loss of isobutylryl moiety replaced by tigloyl moiety. Compound **128** is related to moluccensin G, with the disappearance of 1-*O*-isobutylryl moiety. Compound **129** is composed of phragmalin *ortho*ester. Compounds **120**, **122**, and **123** exhibited antifeedant activities against the third-instar larvae of *B. longissima* [94]. 

The ethanol extract of *X. moluccensis* is rich in limonoids such as khayanolide and xylomolins. Khayanolides are mostly found in the Meliaceae genus *Khaya*, and they are a group of limonoids with the fused A/B ring from octahydro-1*H*-1,6-methanoindene moiety. Krishnolides A–D (**130**–**133**) were isolated from the mangrove seed *X. moluccensis.* Compound **130** has a heptacyclic structure and is similar to thaixylomolin K with the addition of 30-isobutyryloxy moiety, 3-(2-methyl)butyryloxy group, and 8,14-epoxy group, with the loss of C-30 methine moiety, 3-acetoxy group, and a ∆^8,14^ double bond. Compound **131** is similar to **130** with the presence of conjugated ∆^8,9^ and ∆^14,15^ double bonds instead of an 8,14-epoxy group. Compound **132** resembles that of compound **130** with the addition of a ∆^8,14^ double bond replacing the 8,14-epoxy group. Compound **133** is identical to **132,** with the presence of isobutyryloxy moiety and the absence of the 3-(2-methyl)butyryloxy group. Compound **130** showed antivirus activity against the HIV-I virus with an inhibition rate of 79.75 ± 0.77% at 20 μM. The 8,14-epoxy group is responsible for the anti-HIV activity [95].

Ethanol extract of *X. moluccensis* yielded limonoids called xylomolins A1–A7, B1–B2, C1–C2, D–F, G1–G5, H–I, J1–J2, K1–K2, L1–L2, and M–N (**134–162**, respectively). Compounds **134**–**146** are mexicanolides characterized by one double bond or two conjugated double bonds. Compound **145** is a mexicanolide with a C1-O-C8 bridge. Compounds **146**–**151** contain khayanolides with ∆^8,14^ double bonds, compound **152** contains khayanolides with ∆^14,15^, and compound **153** contains khayanolides with ∆^8,9^, ∆^14,15^ conjugated double bonds. Compounds **154** and **155** are limonoids with the presence of (*Z*)-bicyclo[5.2.1]dec-3-en-8-one. Compounds **156** and **157** have 30-ketophragmalins identified by a conjugated double bond at ∆^8,9^ and ∆^14,15^. Compounds **158** and **159** are 8,9,30-*ortho* ester phragmalin, **160** is characterized by azadirone derivatives, and **161** is characterized by andirobin derivatives. Compound **162** showed weak antitumor activity against human triple-negative breast MD-MBA-231 cancer cells (IC_50_ value of 37.7 μM) [96].

Phragmalins 8,9,12-*ortho*esters, called thaixylomolins O–P (**163, 164**); 9,10-*seco* mexicanolide named thaixylomolin Q (**165**); and thaixylomolin R (**166**), a limonoid with 6-oxabicyclo[3.2.1]octan-3-one, were discovered in the seed of *X. moluccensis*. Compound **164** exhibited antitumor against ovarian A2780/T cell and A2780 with an IC_50_ value of 37.5 μM [97].

Limonoids named granatumins L–U (**167**–**175**) were isolated from the ethanol extract of *X. granatum* seed. Compound **167** is closely related to godavarin D with the presence of C8=C30 instead of C8=C14. Compound **162** is identical to compound **167** with the substitution of 3-*O*-2-methylbutanoyl moiety in place of the 3-*O*-tigloyl moiety. Compounds **168** and **169** are correlated with godavarin D with the presence of 3-*O*-Ac moiety and 3-*O*-methacryl instead of 3-*O*-tigloyl, respectively. Compound **170** is closely related to godavarin F with the presence of 3-*O*-Ac moiety in place of the 3-*O*-isobutyryl moiety. Compound **171** is similar to **5** with the addition of 3-*O*-methacryl moiety in the place of 3-*O*-Ac. Compound **172** is identical to **167** with the loss of the C8=C30 bond. Compound **173** is related to **172** with the substitution of 3-*O*-tigloyl moiety by 3-*O*-isobutyryl moiety. Compound **174** is correlated with godavarin K with the presence of 3-*O*-Ac moiety instead of 3-*O*-tigloyl. Compound **175** resembles that of xyloccensin N with the addition of a double bond at C8=C30 [105].

Methanol extract of *X. granatum* seed contained sundarbanxylogranins A–E (**176**–**180**). Compound **176** possesses bicycle[5.2.1]dec-3-en-8-one as a fused ring A/B. Compound **177** contains an 8*α*,30*α*-epoxy ring, and compounds **178**–**180** contain 29-OMe with different orientations. Compound **177** was active against HIV with an IC_50_ value of 23.14 ± 1.29 μM [99].

Granatumin X **(181)** and krishnagranatinins A–I (**182**–**190**) are isolated from the seed of *X. granatum*. Compound **182** is similar to granatumin Y, except for the presence of 3-*O*-isobutyryl moiety instead of the 3-*O*-tigloyl group. Compound **183** is similar to erythrocarpine D, with the substitution of the 3-*O*-tigloyl group in place of the 3-*O*-cinnamoyl group. Compound **184** resembles that of compound **183**, with the addition of the 3-*O*-isobutyryl group replacing the 3-*O*-tigloyl group. Compound **185** is similar to that of **184**, except for the addition of the 9-OH group and substitution of the 3-*O*-acetyl group in the position of the 3-*O*-isobutyryl group. Compounds **186** and **187** are a mixture and are determined in methylation. Compound **188** is similar to that of xylocarpin C with the presence of an acetoxy group in the 6-OH group position. Compound **189** is identical to compound **188** with the loss of the 12-acetoxy group. Compound **190** resembles that of 2,3-dideacetylxyloccensin S with the presence of an acetoxy group instead of the 6-hydroxy group. Compounds **188**–**190** inhibited the NF-κB signaling pathway [98].

Within ethanol extract of *X. granatum* extract, granaxylocarpins A–E (**191**–**195**) were isolated. Compounds **191** and **192** are mexicanolide-type limonoids with a 9,10-*seco* skeleton while compound **193** contains a 1,29-oxygen bridge and 8*α*,30*α*-epoxy ring. Compound **191** is similar to that of xylogranatin B with the loss of tigloyl moiety. Compound **192** resembles that of xylogranatin C with the substitution of C-30 tigloyloxyl moiety in place of C-30 acetoxyl. Compound **193** is identical to xyloccensin L with the addition of C-14 hydroxyl. Compound **194** is closely related to xyloccensin U with the presence of C-6-OH moiety. Compound **195** is similar to compound **194** with the addition of acetyl carbonyl and acetoxyl at C-6. Compounds **191** and **192** exhibited weak toxicity against P-388 murine leukemia with IC_50_ values of 9.3 and 4.9 μM, respectively [100]. 

Thaixylogranins A–H (**196**–**203**) were isolated in ethanol extract of seed of *X. granatum* as compounds **196**–**198**, and **200**–**203** are mexicanolides. Compounds **196**–**199** contain an 8*α*,30*α*-epoxy ring while compounds **200**–**203** possess a C8=C30 bond. Compound **196** is a hexacyclic structure consisting of four ester groups, two carbons double bonds, and a ketone group. Compound **196** is identical to those of granatumin C with the presence of an isobutyryloxy group instead of the 3-tigloyloxy moiety. Compound **197** resembles that of compound **196** with the substitution of two acetoxy groups in the positions of 6-OH and 3-isobutytyloxy. Compound **198** is similar to that of xylocarpin with the presence of a propionyl group instead of a 3-*O*-acetyl group. Compound **199** is identical to moluccensin W with the presence of an ethoxy group instead of a 29-methoxy group. Compound **200** is similar to that of swietenin C with the substitution of 6-acetoxy moiety instead of 6-OH. Compound **201** is identical to that of swietenine acetate with the addition of methacryloyl moiety replacing the 3-*O*-tigloyl moiety. Compound **202** is identical to swielimonoid A with the presence of an acetoxy group in place of the 3-*O*-tygloyl group. Compound **203** resembles that of swielimonoid A with the presence of a 2-methylbutytyloxy group instead of the 3-*O*-tigloyl moiety. Compounds **196**–**203** showed weak cytotoxicity against breast cancer MDA-MB-231 with IC_50_ values of 49.4, 58.3, 53.6, 61.1, 57.9, 44.6, 40.6, and 38.5 μM [101].

Ethanol extract of *X. moluccensis* seed was found to contain stereo-structure limonoids, trangmolins A–F (**204**–**209**). Compounds **204**–**206** possess hexahydro-1*H*-inden-4-one while compound **4** contains hexahydro-2,6-methanobenzofuran-7-one. Compound **208** comprises a hexahydro-2*H*-2,8-epoxychromene while compound **209** is closely related to andhraxylocarpin A [102]. Types of khayanolides, thaixylomolins G–N (**210**–**217**), phragmalins (**218**–**219**)**,** and mexicanolides (**220**–**221**) are isolated with ethanol from *X. moluccensis*. Compound **210** is similar to khayseneganin A with the addition of a C-11 carbonyl moiety, a 30-OH, and a 2-*O*-acetyl. Compound **211** resembles that of compound **210** except for the absence of the C-11 carbonyl moiety. Compound **212** is similar to compound **211** with the replacement of conjugated ∆^8,9^, ∆^14,15^ double bonds by ∆^8,14^ double bonds. Compound **213** is related to compound **212**, with the C-30 hydroxy group being replaced by an ethoxy group. Compound **214** resembles that of compound **212**, with the loss of 30-OH moiety in exchange for C-3 carbonyl moiety and the C-2 acetoxy group. Compound **215** is correlated with compound **214**, with the addition of an ethoxy group. Compound **216** is similar to compound **214**, with the replacement of ∆^8,14^ double bonds by conjugated ∆^8,9^- ∆^14,15^ double bonds. Compound **217** is identical to compound **216**, except for the presence of tigloyloxy moiety. Compound **218** is related to xyloccensin U without 12-acetoxy moiety replaced by a hydroxyl group. Compound **219** is related to compound **218**, with the presence of an acetoxy group instead of the 2-OH moiety. Compound **220** is identical to 2*α*-hydroxymexicanolide with the addition of 6-acetoxy moiety. Compound **221** relates to moluccensin T without 6-OH moiety. Compounds **212**, **214**, and **216** showed anti-H1N1 activities with IC_50_ values of 77.1 ± 8.7, 113.5 ± 8.6, and 121.5 ± 1.5 μM, respectively [103].

Xylorumphiins E–J (**222**–**223**, **225**–**228**) and 2-hydroxyxylorumphhin F (**224**) were isolated from the seed of *X. rumphii*. Compound **222** is composed of six rings, two isobutyryl groups, a methoxycarbonyl, and *β*-substituted furan moiety. Compound **223** is similar to **222** with the presence of a 2-methylbutyryl group instead of isobutyryl moiety at C-30. Compound **224** is closely related to **223**, except for the replacement of the methane group by oxygenated tertiary carbon. Compound **225** resembles that of compound **224**, except for the replacement of the isobutyryl group by the 2-methylbutyryl group. Compound **226** is identical to xylorumphiin A with the presence of acetyl in place of the isobutyryl group. Compound **227** resembles that of **225** with the addition of ∆^14,15^ double bonds. Compound **228** is a phragmalin *ortho*ester. Compounds **224** and **227** exhibited moderate anti-inflammatory activities with IC_50_ values of 24.5 and 3.13 μM [104].

## 5. The Endophytic Fungus in the Mangrove Fruit as a Source of Secondary Metabolites

**Figure 15:**
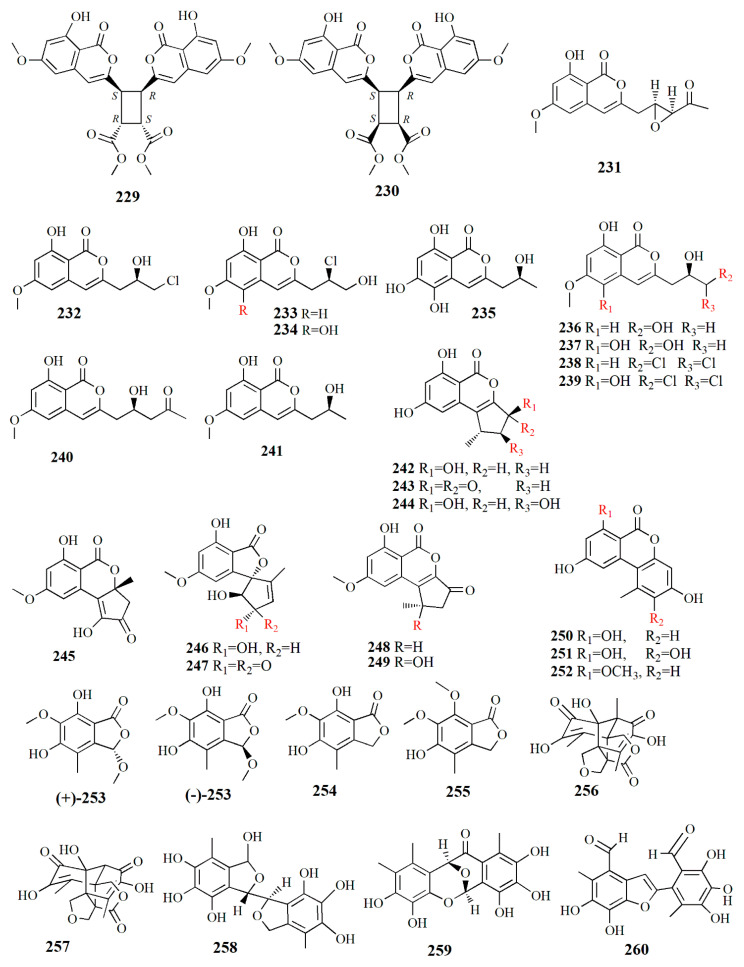
Molecular structure of compounds **229**–**260**.

**Figure 16:**
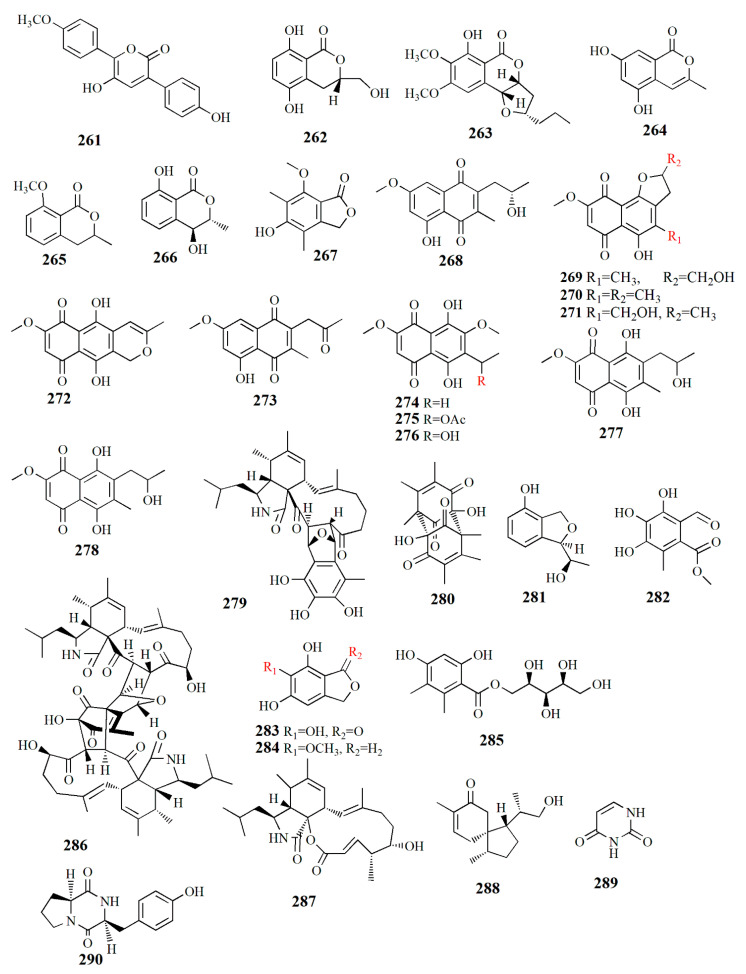
Molecular structure of compounds **261**–**290**.

**Figure 17:**
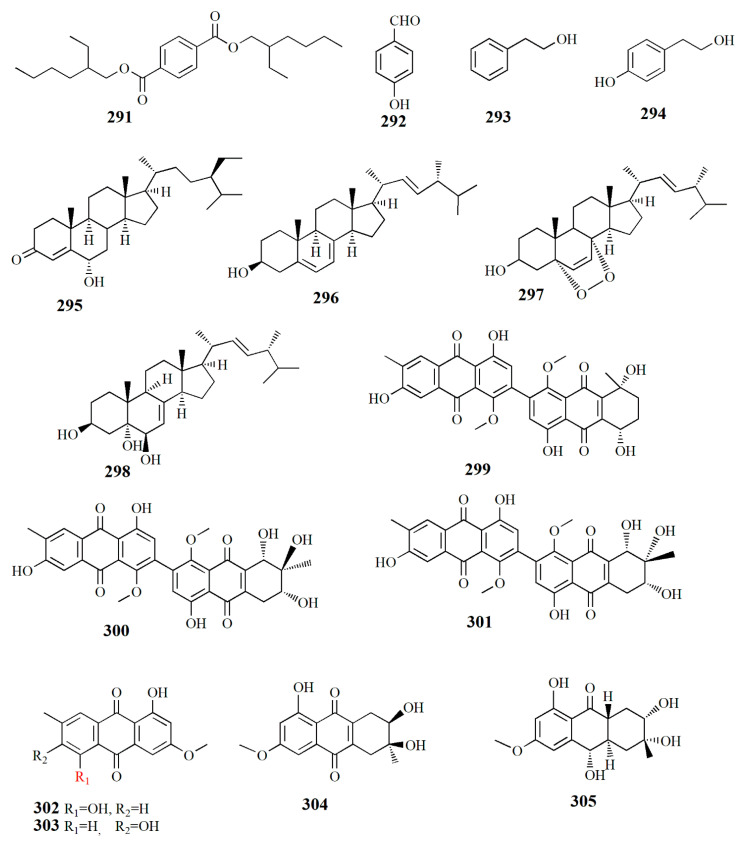
Molecular structure of compounds **291**–**305**.

The mangrove fruit is also a host for fungi. Endophytes inhabit the plant host without triggering problems for the host, sometimes providing nutrients or bioactive metabolites against the phytopathogenic bacteria. Like the fruit, the fungi also biosynthesize treasured phytochemicals for pharmaceutical applications. The isolation of pure fungus and the cultivation of those pure strains led to the discovery of several unique compounds with their bioactivities (Table 4, Figure 15, Figure 16 and Figure 17).

Ten peniisocoumarins were isolated from the fungus *P. commune* from the fresh fruit of *K. candel*. Peniisocoumarin A (**229**), C_28_H_24_O_12_, has the isocoumarins’ nucleus configured by the correlation of H5 at δ_H_ 6.43 ppm to carbon C-5, C-6, C-8, and C-8a, chelated hydroxy group 8-OH at δ_H_ 10.81 ppm to C-8 and C-8a, and methoxy protons 6-OMe at δ_H_ 3.83 ppm to C-6**.** Peniisocoumarin B (**230**), C_28_H_24_O_12_, is similar to compound **229**, with 14 carbons, and 7 non-protonated carbon, 3 sp^2^ methines, 2 sp^3^ methines, and 2 methoxyls. Compounds **229** and **230** are diastereoisomers with the difference in chemical shift of H-9 at δ_H_ 4.07 ppm instead of δ_H_ 4.11 ppm and H-10 at δ_H_ 3.94 instead of δ_H_ 4.00. Peniisocoumarins C (**231**), C_15_H_14_O_6_, was characterized by a hydroxy, two aromatic protons, an olefinic, methoxy group, methylene, and two methines. Peniisocoumarin D (**232**), C_13_H_13_ClO_5_, is similar to the isomarine skeleton of compound **231** with the presence of two methylene groups and one methine group. Peniisocoumarins E (**233**) is an isomer of compound **232** with a similarity in molecular formula; however, it is different in the side chain at C-3. Peniisocoumarin F (**234**), C_13_H_13_ClO_6_, is different from compound **233** in the hydroxy group at δ_H_ 7.85 ppm, the aromatic proton at δ_H_ 6.63 ppm, the olefinic proton at δ_H_ 6.78 ppm, and absence of aromatic proton at H-5. Peniisocoumarin G (**235)**, C_12_H_12_O_6_, resembles that of orthosporin, an isocoumarin with a 2-hydroxypropyl sidechain [115], from fungus *Drechslera siccans*, yet it differs in hydroxy group 5-OH. Peniisocoumarin H (**236**), C_13_H_14_O_6_, is similar to diaportinol yet different in optical rotation and CD spectra. Diaportinol, C_13_H_14_O_6_, was first elucidated from the fungus *Penicillium nagoivense* [116]. Peniisocoumarin I (**237**) is a 5-hydroxylated analogue of compound **236,** and peniisocoumarin J (**239**), C_13_H_12_O_6_, is similar to compound **236,** yet it differs in the presence of the terminal methine group at H δ_H_ 6.21 ppm. Other detected compounds are 3-[-(*R*)-3,3-dichloro-2-hydroxypropyl]-8-hydroxy-6-methoxy-1H-isochromen-1-on1 (**238**); (+)-6-methyl-citreoisocoumarin (**240**), and (+)-diaporthin (**241**). These compounds have been in vitro tested for their bioactivities for metabolic disease, infectious disease, and cancer. In metabolic diseases such as diabetes type 2, *α*-glucosidase is essential in insulin signaling pathways, while in infectious diseases such as tuberculosis, MptpB is secreted into the human host to suppress the immune defense. Compounds **231**, **235**, **237**, and **239** showed strong inhibition toward *α*-glucosidase compared to acarbose at IC_50_ values of 38.1–78.1 μM while **233**, **234**, and **239** show moderate activity of *α*-glucosidase with IC_50_ values of 102.4 and 158.4 μM. Compound **235** showed inhibition to *Mycobacterium tuberculosis* protein tyrosine phosphate B (MptpB) with an IC_50_ value of 20.7 μM. Others showed weak or no inhibitory against MptpB, embryonic kidney HEK293T, breast cancer MCF-7, cervical cancer HeLa, liver cancer HepG2, and lung cancer A549 [106]. 

Altenusin derivatives were isolated from *Alternaria* sp. SK6YW3L from the fresh fruit of *S. caseolaris*. Altenusin derivative 1 (**242**), C_13_H_12_O_5_, contains hydroxyl and carbonyl groups and has two *meta*-coupled aromatic protons at δ_H_ 6.33 and 6.44 ppm, one chelated hydroxyl at δ_H_ 11.31 ppm, one methyl at δ_H_ 1.27 ppm, one methylene at δ_H_ 2.19 and 2.25 ppm, one methine at δ_H_ 3.32 ppm, and one oxymethine at δ_H_ 5.21 ppm. Altenusin derivative 2 (**243**), C_13_H_10_O_5_, is similar to compound **242** with the addition of an *α,β*-conjugated keto carbonyl at δ_C_ 195.7 ppm and the absence of an oxymethine group. Altenusin derivative 3 (**244**), C_13_H_12_O_6_, is similar to **242** with the addition of oxymethine signal at δ_H_ 4.02 ppm and the absence of methylene. Altenusin derivative 4 (**245**), C_14_H_12_O_6_, is similar to compound **243**, with the presence of a carbonyl group at C-8 and methyl group at C-9a. Altenusin derivative 5 (**246**), C_14_H_14_O_6,_ resembles that of compound **247,** except for the absence of *α,β* conjugated keto carbonyl signal and presence of oxymethyl. Compounds **247**–**252** are also isolated in the fungus. Talaroflavone (**247**), C_14_H_12_O_6_, was first isolated from *Talaromyces flavus.* Deoxyrubralactone (**248**), C_14_H_12_O_5_, was characterized by a methoxy group at δ_H_ 3.94 ppm, an aromatic proton, one aliphatic methane, methylene protons, one methoxy group, and one methyl group [117]. Rubralactone (**249**), C_14_H_12_O_6_, has one lactone carbonyl signal at 167.9 ppm, and this lactone carbonyl carbon correlated with the methoxy group and benzene ring [118]. 2-OH-AOH **(250)**, alternariol (**251**)**,** and anternariol methyl ether (**252**) were first discovered in the toxin fungus *Alternaria* [119]. Compounds **243**, **244**, and **250** display strong inhibition of *α*-glucosidase with IC_50_ values of 78.2, 78.1, and 64.7 μM, respectively. Compounds **242** and **249** also show weak inhibition with IC_50_ values of 235.2 and 194.4 μM, respectively. Compounds **245**, **247**, and **251** present moderate inhibition with IC_50_ values of 334, 348.4, and 474.3 μM, respectively. *Alternaria* is member of the ascomycetes family of fungi and is considered a plant pathogen; however, some ascomycetes can be used as a biocontrol of invasive species [107]. 

Endophytic fungus *Epicoccum nigrum* isolated from fresh fruit of mangrove *A. ilicifolius L.* yielded isobenxofuranone monomer and dimer derivatives. Racemic (+)-epicoccone C and (-)-epicoccone C (**253**), C_11_H_12_O_6_, possesses OH groups at δ_H_ 6.53 and 7.55 ppm and one oxymethine carbon at δ_C_ 103.8 ppm. In the separated–purified version, the compound was identified as 3*S* in (+)-1 and *R* in (-)-1. Therefore, compound **253** was recognized as (*S*)-5, 7-dihydroxy-3, 6-dimethoxy-4-methylisobenzofuran-1(3H)-one and (*R*)-5, 7-dihydroxy-3,6-dimethoxy-4-methylisobenzofuran-1(3H)-one. Epicoccone D (**254**), C_10_H_10_O_5_, is similar in the carbon framework of compound **25;** however, 3-OMe in compound **253** is substituted by oxymethylene proton at C-3 in compound **254**. Compound **254** is identified as 5,7-dihydroxy-6-methoxy-4-methylisobenzofuran-1(3H)-one. Epicoccone E (**255**), C_11_H_12_O_5_, is an isobenzofuranone monomer with 2 methoxy protons at δ_H_ 4.03 and 3.85 ppm. Compounds **255** and **254** are similar, the difference being in the replacement of hydroxy groups 7-OH by the methoxy group. Epicolactone A (**256**) is an isobenzofuranone dimer with the molecular formula C_18_H_18_O_8_. The 4-oxymethylenes of compound 256 are shown by the relationship between proton δ_H_ 4.5 and 4.25 ppm; 3.72 and 3.87 ppm; and 3.83 and 3.93 ppm. Compounds **257**–**260** are epicolactone, flavimycins A, epicocconigrone A, and epicoccolide B, respectively. Compounds (+)**-253**, **255**, **259**, and **260** show strong inhibition activity for *α*-glucosidase while compounds (-)-**253**, **254**, **256**, and **258** show moderate activity. The strong activity ranges from 32.3 to 63.3 μM and moderate activity from 130.2 and 252.4 μM. Compounds (+), (-)-253, 258, and 259 had strong oxidant activity with IC_50_ values from 10.2 to 15.3 μM. Compounds **254** and **260** show comparable activity to the positive control; the phenolic group and phenolic hydroxy group are responsible for the antioxidant activity [108].

Allantopyrone E (**261**), C_18_H_14_O_5_, is isolated from rice culture of *Aspergillus versicolor* from *A. marina*. Compound **261** is configured by two aromatic ring systems and *α*-pyrone moiety. The *α*-pyrone ring was established from the correlation of H-4 to C-2, C-3, and C-5 while the *p*-methoxy benzene ring was correlated with C-6. Thus, the compound was named 3,6-diaryl-5-hydroxy-a-pyrone. Compound **261** is derived from phenylalanine via *ter*-phenyl quinone polyporic acid. It presented a cytotoxic effect on HeLa cells with an IC_50_ value of 50.97±1.7 μM [109].

Botryoisocoumarin was detected from *Botryosphaeria* sp. KcF6 from the inner fruit of *K. candel*. 3*S*-5,8-dihydroxy-3-hydroxymethyldihydroisocoumarin or botryoisocoumarin A (**262**), C_10_H_10_O_5_, has a tetrasubstituted hydroquinone ring that is configured by two-*ortho* coupled protons, a methine signal, and methylene signals. The compound is similar to mullein, with the difference in the occurrence of a hydroxymethyl group instead of a methyl group. Compound **262** showed a strong COX-2 inhibitory IC_50_ value of 6.51 μM. COX-2 is an inducible enzyme activated by tumor promoters, endotoxins, mitogens, and cytokines. Monocerin (**263**) has been used as an insecticidal and antifungal, and it was first isolated from the fungus *Microdochium bolleyi* as an endophyte in the herbaceous plant *Fagonia cretica* [120]. Other isolated compounds from the fungus are 3-methyl-6,8-dihydroxyisocoumarin (**264**), 8-methoxymellein (**265**), trans-4-hydroxymellein (**267**), and 5-hydroxy-7-methoxy-4,6-dimethyl phthalide (**268**). Compound **262** inhibited COX-2 activity (IC_50_ value of 6.51 μM) [110].

Naphthoquinone derivatives were successfully isolated from *Talaromyces amestolkiae,* an endophytic fungus of fresh fruit *K. obovata*. Talanaphthoquinone A (**268**), C_15_H_15_O_5_, has one methylene, one oxygenated methine, one methoxy, two methyl groups, two *meta*-coupled aromatics, and one phenolic hydroxy proton. Talanaphthoquinone B (**269**), C_15_H_15_O_7_, is similar to compound 268, with the difference in two quinone carbonyls at C δ_C_ 177.6 and 190.1 ppm. The extract also contains other compounds, such as: anhydrojavanicin (**270**), 2,3-dihydro-5-hydroxy-4-hydroxymethyl-8-methoxy-2- methylnaphtho[1,2-b]furan-6,9-dione (**271**), anhydrofusarubin (**272**), 2-acetonyl-3-methyl-5-hydroxy -7-methoxy-naphthazarin (**273**), 6-ethyl-2,7-dimethoxyjuglone (**274**), 6-[1-(acetyloxy)ethyl]-5-hydroxy-2,7-dimethoxy-1,4-naphthalenedione (**275**), 5-hydroxy- 6-(1-hydroxyethyl)-2,7-dimethoxy-1,4-naphthalenedione (**276**), solaniol **(277**), and javanicin (**278)**. All of these compounds (**268**–**278**) showed strong inhibition against lipopolysaccharide (LPS)-activated NO production in the murine macrophage cell line (RAW 264.7 cells) with IC_50_ values of 3.9, 49.7, 16.0, 22.6, 11.2, 5.2, 14.4, 7.7, 1.7, 7.5, 15.5, and 5.6 μM, respectively. Compound **275** reduced the mRNA expression of interleukin (IL)-6, IL-1B, the pro-inflammatory cytokines tumor necrosis factor (TNF)-*α*, cyclooxygenase-2 (COX-2), and inducible nitric oxide synthase (iNOS). Those proteins are responsible for metabolic action during inflammation. The macrophage and monocyte produce the overexpression of pro-inflammatory cytokines such as IL-6, IL-6B, TNF-α, and also prostaglandin E2 (PGE2); meanwhile, NO is produced by inflammatory factors such as COX-2 and iNOS. Overproduction of those compounds leads to inflammatory and cell disease. In normal conditions, inflammation is a response to tissue injury and infection. However, longer inflammation may cause diseases such as septic shock syndrome, a neurodegenerative disorder, or arthritis [111].

Fungus *Mycosphaerella* sp. from fruit *Bruguiera* gave compounds with *α*-glucosidase inhibition. Asperchalasine I (**279**), C_33_H_41_O_7_N, is configured by the presence of epicoccine moiety and cytochalasan moiety. Dibefurin B (**280**), C_18_H_20_O_6_, has carbonyls and a hydroxyl group. The phthalan ring present in epicoccine derivatives (*R*)-9-((*R*)-10-hydroxyethyl)-7,9-dihydroisobenzofuran-1-ol (**281**), C_10_H_12_O_3_, while aldehyde group and methyl ester appear in 2-methoxycarbonyl-4,5,6-trihydroxy- 3-methyl-benzaldehyde (**282**), C_10_H_10_O_6_. Other compounds were also isolated from the extract, such as epicoccone B (**283**), 1,3-dihydro-5-methoxy-7-methylisobenzofuran (**284**), paeciloside A (**285**), asperchalasine A (**286**), and aspochalasin I (**287**). Strong *α*-glucosidase inhibitory was shown by compounds **279** and **286** with IC_50_ values of 17.1, 26.7, and 15.7 μM while compounds **279**, **282**, and **284** had antioxidant activity with EC_50_ values of 16.3–85.8 μM [112]. 

The investigation of the fungi extracts of *Pseudofusicoccum* sp. J003 from fruit *S. apetala* led to the discovery of compounds, i.e., sesquiterpenoid acorenone C (**288**), alkaloid uracil (**289**), alkaloid cyclo-(*L*-Pro-*L*-Tyr) (**290**), phenolic bis-(2-ethylhexyl) terephthalate (**291**), phenolic 4-hydroxybenzaldehyde (**292**), phenolic 2-phenylethanol (**293**), phenolic 4-hydroxyphenethyl alcohol (**294**), steroid estigmast-4-en-6B-ol-3-ona (**295**), steroid ergosterol (**296**), steroid ergosterol peroxide (**297**), and steroid cerevisterol (**298**). Compound **288** showed AChE inhibitory activity with an inhibition rate of 23.34% at 50 μM. Compound **296** also showed an inhibition rate of 72.89% NO production at a concentration of 25 μM and human tumor cell lines HL-60 and SW480 with inhibition rates of 98.68% and 60.40%, respectively [113].

The fungus *Alternaria* sp. ZJ9-6B from the fruit *Aegiceras corniculatum* yielded at least seven compounds. Alterporriol K (**299**), C_32_H_26_O_11_, is constructed by a tetrahydroanthraquinone unit and an anthraquinone unit. Alterporriol L (**300**), C_32_H_26_O_12_, is similar to compound **299** with the addition of an oxymethine group and a hydroxyl group. Alterporriol M (**301**), C_32_H_25_O_12_, is identical to **300**; both compounds are epimers, diastereomers, and atropisomers. Other isolated compounds from the extract are physcion (**302**), marcrospin (**303**), dactylariol (**304**), and tetrahydroaltersolanol B (**305**). Compound **299** showed cytotoxicity against human breast cancer cell lines MDA-MB-435 (IC_50_ = 26.97 μM) and MCF-7 (IC_50_ = 13.11 μM). Compound **300** showed cytotoxicity against MDA-MB-435 (IC_50_ = 13.11 μM) and MCF-7 (IC_50_ = 20.04 μM) [114].

Two endophytic fungi, *Cladosporium tenuissmum* and *E. nigrum*, were isolated from the fresh fruit of *S. apetala*. The ethyl acetate extract of *C. tenuissimum* demonstrated antibacterial activity against *E. coli, Micrococcus luteus, P. aeruginosa,* and *S. aureus,* while its methanolic extract depicted antimicrobial activity against *E. coli, M. luteus, P. aeruginosa, S. aureus,* and *C. albicans.* Ethyl acetate extract of *E. nigrum* showed antibacterial activity against *M. luteus*, *P. aeruginosa*, and *S. aureus*, while the methanolic extract showed antibacterial against *E. coli*, *M. luteus*, *P. aeruginosa*, and *S. aureus* [121]. 

## 6. The Mangrove Fruit in the Intermediary Stage of Processed Food

The supply-chain problem challenges the dominancy of wheat flour as a daily ingredient. Wheat grows worldwide as a major cereal source; however, the demand has increased exponentially. Wheat flour is crucial for bakeries and many household products [122]. Other emerging edible flours are required to supplement the demand with cost-effective and fortification functions. The flour from fruit is reliable in solving the problem of the world’s wheat stock [123]. Among the fruit, the flour of mangrove fruit is a proper candidate due to its richness in protein, carbohydrate, alkaloid, flavonoid, steroid, and other metabolites [124]. Many coastal communities utilize mangrove fruit flour for biscuits, flatbread, and crackers.

Usually, the mixture of mangrove flour and other ingredients produces unique characteristics. For instance, the mixture of wheat flour and *B. gymnorrhiza* flour (BGF) generates different functional and rheological properties, such as changes in the peak time, setback viscosity, final viscosity, and breakdown viscosity. The dominant factor affecting those properties is the water content [125,126]. The BGF is different from wheat flour in its amylopectin and amylose content. Flour with higher amylopectin conserved water more than flour with lower amylopectin [127]. BGF contains water-soluble fractions such as tannin, while tannin reduces the hydrophobicity of flour protein (gluten). Thus, the addition of BGF increases the water absorption capacity. During the production of mangrove flour, the size of particles in the final flour product is essential [128]. Flour with a finer particle size is better in making contact, frictional forces, and cohesive force than flour with a coarse particle size [129]. 

In the food innovation process, many types of wheat are mixed to offer a better product with acceptable texture and nutrition content. The mangrove fruit flour can be mixed with other types of flour such as sweet potato flour and maize starch, which affect the peak viscosity. The pasting temperature of the mixture of mangrove fruit flour and sweet potato flour exhibits no significant difference compared to the single-source pure four. The peak viscosity is the most sensitive parameter affected by the addition of mangrove fruit flour. During the mixture of mangrove flour and potato starch, the peak viscosities are significantly correlated with the breakdown viscosities. Meanwhile, the addition of the mangrove fruit reduces the viscosity of waxing maize starch. The peak viscosity is the maximum viscosity during cooking and affects the paste strength in response to gelatinization [130]. The pectin content within the mangrove flour is the substance determining the viscosity properties since pectin gives gelling properties to the mixture [131]. Pectin is the soluble dietary fiber in mangrove fruit, and lignin, hemicellulose, and cellulose are insoluble dietary fibers. Insoluble dietary fibers usually decline the sensory quality of the food, while soluble dietary fibers improve it. Thus, the ratio between insoluble and soluble dietary fibers is important in maintaining the quality of the food product [132].

Low viscosity indicates higher resistance to retrogradation, and the addition of mangrove fruit enhances the retrogradation. Retrogradation is a step wherein the gelatinized starch forms an ordered configuration by cooling to increase the crystallinity to create rigidness and firmness of the product [133,134]. The addition of mangrove can be used for a product with a high final viscosity, such as pudding. On the contrary, high viscosity is unfavorable for baked food due to high breakdown viscosity representing a lower ability to withstand heating and shear stress [135]. The mixture of mangrove flour and wheat flour provides no significant improvement in viscosity due to the interaction of starch and non-starch content (protein–fiber) of the wheat flour to increase swelling. The breakdown viscosity of the mixture between mangrove fruit flour and wheat flour is positively related to the peak viscosity. The starch content of mangrove four is negligible, the change in pasting viscosity is derived by the swelling of pectin, and the pectin does not bind or withdraw water from the starch [136]. To catch the whole scope of the safe usage of the mangrove fruit flour, the animal test and preliminary test on food making are investigated [132].

*B. gymnorrhiza* flour (BGF) is rich in pinitol, a strong antioxidant to restrain ulcerative colitis. Ulcerative colitis is an inflammatory abdominal disease in the rectum and colon [137]. Ulcerative colitis is related to a limited oxidative response in the intestine [138]. The administration of the flour to male BALB/c mice maintains the bodyweight while declining the histological score, improving colon pathologic variation, restoring the colon length, and alleviating inflammatory mediators such as IFN-γ, IL-1B, L-6, and MDA levels. The BGF up-regulates the protein (mRNA) level of NQO1, HO-1, GCLM, GCLC, and nuclear Nrf2 while inhibiting the cytosolic Nrf2 and protein expression of Keap1. NF-E2-related factor 2 (Nrf2) is responsible for the oxidative stress response either due to endogenous or environmental stress, and it is regulated by Kelch-like ECH-associated protein 1 (Keap1) [139]. The activation of the Keap1/Nrf2 signalling pathway is powerful in the therapeutic procedure of ulcerative colitis [140]. Nrf2 antioxidant enzymes to treat ulcerative colitis are the glutamate-cysteine ligase catalytic subunit (GCLC), NAD(P)H quinone dehydrogenase 1 (NQO1), hemeoxygenase-1 (HO-1), and glutamate-cysteine ligase modifier (GCLM). Moreover, BGF promotes the growth of probiotics such as *Lactobacillus*, *Anaerotruncus*, and *Bifidobacterium* in the gut while restraining pathogenic bacteria *Streptococcus* and *Bacteroides*. The balance of the flora community restrained the abnormality in intestinal mucosa and then minimized the oxidative stress. Oxidative stress is a common inflammatory disease caused by pathogenic infection. BGF demonstrated the DPPH scavenging activity with an IC_50_ value of 20.45 μg/mL [141].

The administration of *R. mucronata* flour shows hypoglycemic activities and reduces the blood glucose of male Wistar rats. Even though the effect is incomparable to a standard drug, with glibenclamide, the response in blood and urine is similar. The antidiabetic activity is due to the dietary fiber content and the bioactive compound [142]. Ripe fruit flour of the mangrove declines the blood glucose in a dose-dependent manner while maintaining the bodyweight of streptozotocin-induced mice. Diabetic organisms favor losing the bodyweight since they burn fat rather than glucose. The flour contains 3.5% protein, 0.78% fat, 819 ppm tannin, and 46.1% dietary fiber including 38.6% insoluble fiber and 7.5% soluble fiber. The flour is low in fat and protein while high in carbohydrate and insoluble fiber. The high dietary fiber with soluble fiber helps to reduce plasma lipid in type 2 diabetes, reduce hyperinsulinemia, and maintain blood glucose levels [142].

The administration of the mixture of fruit flour with sweet potato, arrowroot starch, taro starch, potato starch, and cassava starch in the Wistar rat model produces different outcomes. The performance of each mixture is assessed by considering the glycemic index. The glycemic index of food determines the significance of carbohydrates in the diet and relates to the effect on blood glucose [143]. Food with a high glycemic index escalates the metabolic condition [144]. The best mixture is 20% mangrove fruit and 80% taro starch, resulting in a 7.39 glycemic load, 48.83 glycemic index, 2.72% crude fiber, 15.55% fat, and 2.58% protein. Food with a low glycemic index helps to restrain appetite, improving the glucose and fat level [145].

## 7. The Prospect of Mangrove Fruit in the Functional Food Development

Referring to sustainable development growth proposed by the United Nations, a new strategy is required to solve the world’s hunger either by a physical, political, or economic approach. In the physical aspect, the concepts of bioeconomy in the food industry arise by utilizing the material from renewable resources [146]. Fruits such as mangrove fruit come as an option with their richness of phytochemicals. However, it is worth noting that the exploration of mangrove fruit should be reflected in the conservation strategies in each region. The population of mangrove plants is decreasing based on the IUCN, viz., *A. marina* and *B. gymnorrhiza*. The massive exploitation of the fruit has to be in balance with the plantation and harvesting strategies. Nevertheless, the campaign on the usage of mangrove fruit as functional food will hopefully improve the communities’ understanding of the priceless value of the fruit and the mangrove forest itself. With the increasing awareness of the community of the mangrove fruit value, the conservatism effort will, naturally, increase. Studies on the food products derived from the mangrove fruit are intensively directed to date. Many processed foods derived from mangrove fruit are patented (Table 5), including ingredients [147,148,149], beverages [150,151,152], and even synthetic rice [153]. Though the market study still needs to be carried out, the literature depicts the potency of mangrove fruit to be processed into unique food. 

**Figure 18:**
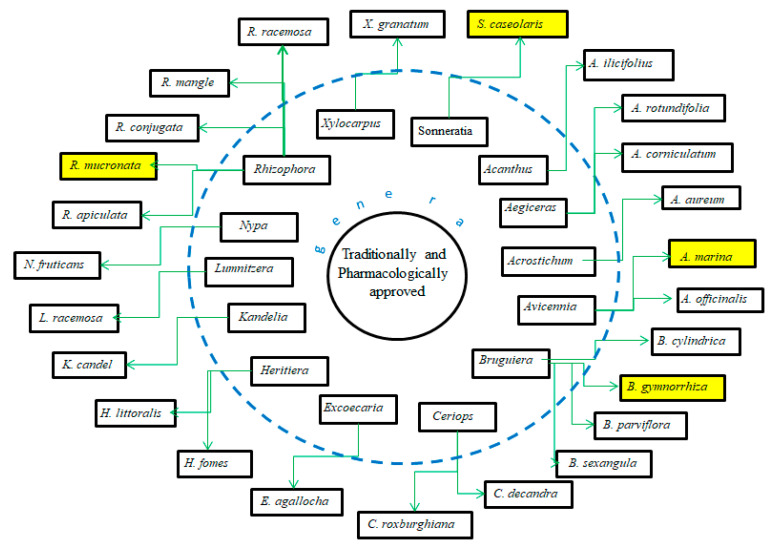
Species of mangrove that are traditionally and pharmacologically proven for their ethnomedical properties; the yellow boxes are species common for traditional food. Modified from [32,128,141,165,166].

About 27 species of mangrove are approved traditionally and pharmacologically for folklore medicine [32]. The medicine is derived from parts of mangrove such as the stem, bark, leave, root, or fruit. However, fruit from limited species are developed into traditional food (Figure 18), i.e., *B. gymnorrhiza, R. mucronata*, *S. caseolaris*, and *A. marina*. Juice-making is a simple and conventional way of processing fruit, a process that mainly involves pulping the fresh fruit [160]. The juice, which involves a heating process from ripened fruit *S. caseolaris,* contains 65 mg of vitamin C [161]. Heating is sometimes required to prepare the juice for sterilization purposes; however, the heating should be for the proper duration. The longer time of heating is highly likely to decompose metabolites. During the heating, the total polyphenol level increases from 0 minutes to 20 minutes and declines afterwards. Polyphenols are stored in the pectin network or cell wall, then the heating assists in breaking the cell wall [162]. However, boiling reduces the vitamin C from 187.46 to 151.92 mg/100 g and protein content from 52.78% to 38% [163]. Thus, the pulp of mangrove fruit *S. caseolaris* is developed into a fruit drink with that is high in phenolic compounds [164].

The juice of *S. caseolaris* is traditionally applied as an antiseptic and astringent to stop hemorrhages and treat coughs. The phenolic compound and antioxidant properties of unripe fruit are higher than ripe fruit. In the ripe fruit, the compound is degraded due to the biosynthesis pathway controlled by enzyme expression, environmental factors, and genetics. The antioxidant activity of the juice has a strong correlation with the total phenolic, flavonoid, and carotenoid content [39]. The juice of *Barringtonia racemosa* showed antioxidant and *α*-glucosidase inhibition activities, while the juice of *Thespesia populnea* showed antihyperglycemic- and hypoglycemic-induced diabetes in the rabbit. Those fruits contain polyisoprenoids such as dolichols and polyprenol. The dolichols content differs among fruits, i.e., *B. racemosa* is comprised of C50–C110 and *T. populnea* is comprised of C65–C100. Polyprenol is only found in *T. populnea* (C55–C90). Dolichols are sugar-carrier lipids in the N-glycoprotein biosynthetic and GPI-anchored protein [167].

Other biscuits are made from the fruit of two mangrove plants, *S. caseolaris* and *B. gymnorrizha*, with antidiabetic and anticholesterol activity in male Wistar rats. The blood glucose level declines to 52.22% (109.91 mg/dL) as a result of the hypoglycemic effect of the bioactive compound and dietary fiber. The mangrove fruit flour from *S. caseolaris* has 63.70% dietary fiber and showed glucose inhibition [168]. The biscuit stays in a shorter time in the intestine than wheat flour biscuit, reducing glucose absorption. In diabetic rats, the bodyweight decreased by up to 6.32% due to the damage to B pancreas cells from alloxan induction. This stimulates the production of insulin and the glucose to be absorbed into the cell. The feeding of biscuits increased the body weight by up to 11.13%, showing a response to the enhancement of peripheral insulin sensitivity. The feeding reduced the cholesterol by 47% and LCL cholesterol, HDL cholesterol, and triglyceride by 50.47%. Dietary fiber controls the absorption and metabolism of lipid and glucose [169]. 

Another innovation is the food bar from local materials, and it provides advantageous dietary intake during an emergency such as flooding or an earthquake. As an emergency arises, convenient food with sufficient nutrient content is required. The availability of edible raw materials in the surrounding environment is important. The food bar made of mangrove fruit flour, soybean flour, and broccoli flour is beneficial in minimizing malnutrition in elderly people. The consumption of the food bar increases the bodyweight, and it is efficient in providing macronutrients [170].

Kombucha herbal from mangrove fruit *R. mucronata* showed antidiabetic and antioxidant activity by *α*-glucosidase inhibition with an IC_50_ value of 33.95 ppm. Kombucha is a traditional drink with a sweet-sour taste from the fermentation of a mixture of sugary herbs or tea by microbial tea starters [171]. The starter microorganism forms an elastic layer called "nata" or "tea mushroom" on the herb’s surface and tea liquid on the bottom. The microorganism is a consortium of bacteria and fungi. The bacteria are from the genera *Acetobacter* and *Gluconobacter*, while the yeast is *Mycoderma*, *Mycotorula*, *Pichia*, *Kloeckera*, *Brettanomyces*, *Torulospora*, *Candida*, *Schizosaccharomyces*, *Saccharomycodes*, *Zygosaccharomyces*, and *Saccharomyces* [172]. The beverage has a sour and sweet taste based on the type of starter, and the flavor comes from tannin transformation [173]. Kombucha herbal tea is famous for its antioxidant and antimicrobial activities [174]. A positive correlation between the tannin content with antidiabetic and antioxidant activity is observed in kombucha from mangrove fruit. The kombucha is prepared with 10% sugar addition and fermentation within 14 days. During the fermentation, the kombucha produces 19679.82 mg GAE/100g of total phenolic acid [175]. Mangrove fruit is also processed into other drinks such as tea and coffee. Tea and coffee from mangrove fruit contain quercetin-3-*O*-(2”-*O*-glucopyranoside)-galactopyranoside, dodecanoic, quercetin-3-*O*-galactopyranoside and *L*-alanine. Both extracts of tea and coffee from mangrove fruit demonstrate high antioxidant activities, i.e., tea mangrove with an IC_50_ value of 4.13 μg/mL and coffee mangrove with an IC_50_ value of 5.25 μg/mL [166].

*S. caseolaris* extract can be added as an additive or perseverance for palm olein by restraining lipid oxidation. Palm olein is vegetable oil as a fraction of palm oil after crystallization. Palm olein has low oxidative stability due to high (up to 55%) unsaturated fatty acid, mainly oleic acid (C18:1n9) and linoleic acid (C18:2n6). It is a challenge to maintain the quality of the oil for marketing purposes. The main problem is oxidation, leading to discoloration, an offensive odor, destructive vitamins, enzymes, and other nutrients. Minimizing the oxidation, the antioxidant is added, and a natural antioxidant is preferable due to its lower toxicity, even if it is more expensive. The antioxidative activities of the mangrove fruit extract are related to the total phenolic compounds. With the addition of mangrove fruit extract, the storage period increases with the increased formation of primary lipid oxidation product (hydroperoxide) [176].

## 8. The Possible Strategic Development for Mangrove Fruit as Functional Food Material

The mangrove fruits, indeed, show great potency in functional food development given their richness of phytochemicals and their potential for improvement as processed food. Traditional food based on edible mangrove fruit has long been applied in some South Asian and Southeast Asian countries. However, the search for the mangrove fruit candidate as functional food should well address the safety aspects regarding the fruit’s chemical content since some fruits may not be edible. Semi-mangrove *Excoecaria agallocha* L. and mangrove *Heritiera littoralis* are notorious examples of mangrove species producing poisonous compounds [177,178]. Even though the poison is merely produced in the bark of the mangrove, the phytochemical activities of the fruit need to be investigated. The latex from *E. agallocha* bark causes temporary blindness [177]; the leaf extract is used as dart poison and fish poison in many countries including Goa, Sarawak, and New Caledonia [179]. The latex of *H. littoralis* is also used as spearhead and arrowhead poison and fish poison [178]. Investigation is also required for other mangroves.

Concurrent with the poisonous latex from its bark, the seed of *E. agallocha* and *H. littoralis* is acknowledged for its ethnomedicinal properties. The bark of *E. agallocha* is rich of diterpene such as *ent*-3,4-seco-16α-hydroxyatis-4(19)-en-3-oic acid, which possesses anti-microfouling activity against *Pseudomonas pseudoalcaligenes* (EC_50_ value of 0.54 ± 0.01 ppm) [179]. *E. agallocha* is an important mangrove plant in East and Southeast Asia as a traditional remedy for dermatitis, conjunctivitis, epilepsy, and toothache. Various triterpenoids, flavonoids, and tannins are isolated from the species [180]. Meanwhile, the seed of *H. littoralis* is edible and consumed for traditional medicine in Andaman Island and Nicobar for treating diarrhea and dysentery [178]. Unfortunately, there are limitations in the literature for the fruit of both species.

It is worth noting that evaluating the toxicity and hazardous compounds within the mangrove fruit is important. On the phytochemical aspect, the compounds from mangrove fruit and other parts of the mangrove are toxic and can be utilized for larvicidal, acaricidal, and pesticidal applications. The leaf extract of *R. mucronata* has larvicidal potential against mosquito larvae, i.e., *Aedes aegypti* (LC_50_ = 0.11 mg/mL), *Culex quinquefasciatus* (LC_50_ = 0.13 mg/mL), and *Anopheles stephensi* (LC_50_ = 0.34 mg/mL). The active compounds with applications as larvicidals are phenols, tannins, saponins, flavonoids, and quinones [181]. The fruit of *C. manghas* shows acaricidal activity against female adults of *Panonychus citri* (LC_50_ = 3.39 g/L) [55]. The mangrove forest also has a remarkable area containing pollutants, including solid particles such as micro/nanoplastics [182], organic pollutants (e.g., PAHs, PCBs, and other POPs), and metals [183,184,185,186]. Those pollutants potentially accumulate within the mangrove plant [187]. Therefore, the evaluation of those hazardous compounds within the mangrove fruit is coveted prior to their development into functional food.

**Figure 19:**
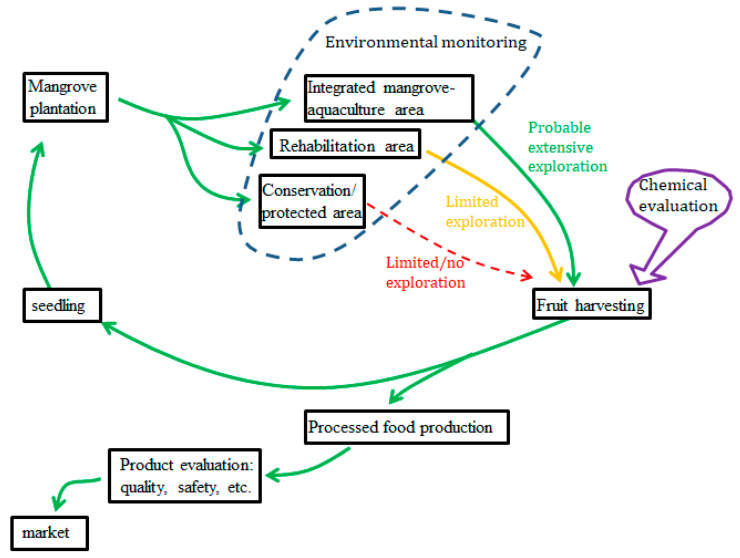
The proposed strategy development for utilizing mangrove fruit for functional food production.

It is acknowledged that mangroves play a crucial role in aquatic environments. The fruit of mangroves provides nutritious food for animals such as birds, reptiles, and mammals living in the mangrove forest [188]. The harvesting of mangrove fruit for human consumption in conservation or protected areas has to consider the natural capacity to preserve the habitat and its inhabitants. Thus, when it is widely accepted by the society, other approaches to support the mangrove fruit utilization for human food are demanded. Though the mangroves are not considered cultivated plants, there are rehabilitation and mangrove reforestation success stories in many places. The *B. gymnorrhiza* plantation in south Florida in the 1940s is an excellent example in which the population growth rate reached 5.6% per year [189]. These successes reflect the potency of mangrove to potentially be cultivated for agricultural purposes (Figure 19). To cope with the mangrove fruit demand for human consumption, the fruit can be collected from man-made areas. The man-made area is divided into at least two actions: (1) a sole man-made mangrove ecosystem for agricultural purposes, and (2) the integrated mangrove-aquaculture area. The integrated mangrove-aquaculture or silvofishery are applied in many countries such as Indonesia, Vietnam, and Nigeria [190,191,192]. In Indonesia and Vietnam, the mangrove is planted in the water canal between platforms [193]. From these man-made areas, the mangrove fruit and other mangroves forest products can be extensively explored, even though the justified management should be applied. The type of planted mangrove can be selected considering the ecological aspects. 

The harvested mangrove fruit has been subjected to chemical analysis to investigate the safety or hazardous chemical of the fruit [194]. The common phytochemical compounds from mangrove fruit, in high concentrations, expose a threat to human health; therefore, the amount is reduced during processing. A biscuit can be made from the mixture of mangrove fruit *B. gymnorrhiza* flour and soybean flour with a ratio of 7:2. The manufacturer modifies the chemical content during food processing by performing some treatment. Some mangrove fruit contains a high level of HCN and tannin that are unhealthy for the human body. Boiling and the addition of ash during biscuit production reduce the amount of HCN and tannin. The boiling process reduces the HCN and tannin content, while the addition of ash inhibits the metabolism rate of tannin. Tannin is decomposed to glucose, and gallic acid during the heating and rubbing ash absorbs the tannin. The addition of mangrove fruit increases the fat and protein contents to relatively higher amounts compared to commercial plain flour cookies [165]. Thus, the evaluation of chemical content from the mangrove fruit for functional food is commenced after the fruit harvesting and food production. 

## 9. Current Studies of Mangrove Fruit in Middle East

Using the Landsat data, it can be seen that the mangrove forest in the Arabian Gulf and the Gulf of Oman increased from 1977 to 2017. The mangrove afforestation effort supported by government regulation shows a positive outcome in each of the Gulf countries during 1977–2017, i.e., Iran (from 4735 ha to 9403 ha), United Arab Emirates (from 1327 ha to 7926 ha), Oman (from 206 ha to 530 ha), Arabian Gulf of Saudi Arabia (from 97 ha to 837 ha), Qatar (from 16 ha to 1002 ha), and Bahrain (from 31 ha to 48 ha) [195]. 

Mangrove forest in the Red Sea, Kingdom of Saudi Arabia, grows in a narrow or broad forest in coastal, nearshore, or offshore islands. Four species—*A. marina*, *B. gymnorrhiza*, *C. tagal*, and *R. mucronata*—are recorded along the Red Sea part of Saudi Arabia [196]. The mangrove forest in the Red Sea part of Saudi Arabia is mostly composed of *A. marina* with a small co-existence of *R. mucronata*. The mangrove forest is in a patchy pattern rather than continuous distribution. The mangroves are slow growers in the area due to environmental and anthropogenic stress such as pollution, poor soil texture, high salinity, and low precipitation. The Red Sea in the Arabian Peninsula is characterized as an arid and oligotrophic area [197]. The Landsat imaginary identified the slight increase by about 0.29% per year of mangrove stands from 1972 to 2013 due to rehabilitation efforts [198]. 

The rehabilitation effort of mangrove forests in the Red Sea in the Arabian Peninsula mainly introduce *A. marina* since the species is salt-tolerant up to 70 ppt, and the survival rate of the plantation reached 39% during two years of the program [199]. The growth of *A. marina* in the Red Sea is affected by the nitrogen limitation. The tallest trees are observed in the southern part of the Red Sea in the Arabian Peninsula, while the smallest is recorded in the central of the Red Sea. At the same time, the intermediate high is documented in the northern part of the Red Sea. The distribution follows the nitrogen concentration [200]. *A. marina* allocates its biomass belowground more than aboveground to cope with the nutrient limitation and hypersaline, anoxic, soft, and unstable sediment. The allocation of this belowground biomass is more significant in the arid area. The belowground biomass of *A. marina* in Shuaiba on the Saudi Arabian Red Sea coast is greater than in East Africa [201]. The photoperiods, temperature, humidity, and salinity affect the reproduction of *A. marina* in the central Red Sea of Saudi Arabia [202]. *A. marina* occupies 135 km^2^ of the coastal area. The forest is a low organic-carbon C_org_ sink due to extreme conditions such as the inclining soil respiration rate, the declining growth rate of mangrove, high temperature, poor nutrition, and low rainfall [203]. Mangrove forests in Yanbu (0.39 t C/ha) store more organic carbon than Umluj (0.12 t C/ha) and Ar-Rayis (0.11 t C/ha). Mangrove forest in Yanbu is a protected area, while the others are non-rehabilitation and non-conservation areas [204].

*A. marina* and *R. mucronata* are two species of mangrove discovered in the Red Sea part of the Egyptian coastal area [205], while *A. marina* is the only mangrove that grows in south Sinai, Gulf Aqaba, Egypt [206]. Mangrove on the Egyptian Red Sea coast is dominantly monospecific with *A. marina.* The variation in *R. mucronata* occurs on the Egyptian–Sudanese border coexisting with the dominant *A. marina*. The mangrove occupies 525 ha spreading in 28 locations on the Egyptian Red Sea coast [207]. On the Egyptian Red Sea coast, the growth of *R. mucronata* is higher when associated with *A. marina* compared with its pure stand. The soil composition (e.g., Na^+^, K^+^, Ca^2+^, Mg^2+^, Cl^−^, and CaCO_3_), morphological characteristics, and tidal inundation determine the distribution pattern of mangrove species. The root system of both mangroves affects the survival rate [208]. The mangrove on the Red Sea Yemeni coast is denser than mangrove in the Red Sea southern Saudi Arabia part since they receive less anthropogenic stress. They grow in intertidal, lagoon, and estuarine areas, covering around 1610 ha. As in other parts of the Red Sea, the mangroves grow in a patchy pattern instead of in a continuous distribution [209]. *A. marina* is the dominant mangrove species but co-exists with small *R. mucronata* in Kamaran Island, Yemen. The mangrove in the offshore island is denser than the coastal area of the Arabian Peninsula [210].

In the Arabian Gulf, *A. marina* is also the dominant species. However, the growth of *A. marina* in the western part of Arabia Gulf is slow due to harsh environmental conditions and pollution, e.g., from the Gulf War oil spill in 1991 [211]. The plantation of *A. marina* in Qatar shows positive feedback after 23 years of the plantation program, with a density increase from 1100/ha to 2100/ha. The natural habitat covers 797.6 ha; the planted area is 182.9 ha on the east coast, while it is only 1 ha on the west coast. However, the natural mangrove is higher (1–6 m) compared to planted mangrove (1–3 m) [212]. Meanwhile, the mangrove area in Oman covers 0.33% of the coastline [213]. An impressive increase in mangrove forests is observed in the United Arab Emirates: mangrove forest in Khor al Beida is undisturbed and is in a natural condition [214].

The mangrove forest in the Middle East is relatively less dense with has fewer recorded species than in other parts of the world. The local community utilizes the mangrove plant as traditional medicine, and several studies on the bioactive compounds are also explored (Table 6). Extract of *A. marina* leaf from Safaga, Red Sea, Egypt, possesses antibiofilm formation against *Pseudomonas fluorescens* by preventing initial cell attachment at an IC_50_ value of 42.0–45.8 mg/mL [215]. Ethanol extract of *A. marina* from Saudi Arabia possesses antidiabetic activity by reducing the blood glucose level; methanolic extract of the aerial root shows antihyperglycemic properties. Aqueous extract of *R. mucronata* bark presents antihyperglycemic and hypoglycemic activities. Water extract of *A. marina* leaf in Iran also shows antidiabetic properties by reducing the serum levels in the liver [216]. The bark of *A.marina* from Jazan, Saudi Arabia, is traditionally used to treat diabetes and smallpox, to induce infertility in women, and as a pruritic [217]. However, the fruits’ development into functional food is limited.

## 10. Conclusions

Through various profound investigations, valuable nutrition data and many classes of metabolites with bioactive properties from the mangrove fruits have been discovered. The proximate of mangrove fruit differs regarding the type of the fruit and the crude extract possesses antioxidant activities related to the amount of total phenolic content. The extract of mangrove fruit to animal models exhibits antioxidant, anti-atherosclerosis, antimicrobial, anti-diabetic, and hepatoprotective properties. The mangrove fruit is also rich in limonoids, a group of terpenoid compounds that show antioxidant, antidiabetic, and anticancer properties. Interestingly, the seed and symbiont, endophytic fungus, are also a source of secondary metabolites with various bioactivities. Observed deeply, some compounds found in the seed are similar to those of compounds from the fruit, such as the class of xyloccensins; however, the class of compounds from the endophytic fungi is different from them both. It is suspected that the biosynthetic pathway of the compounds in the fruit resembles that of the seed, yet the endophytic fungi may possess distinct biosynthetic pathways.

The mangrove fruits exhibit an excellent prospect of transforming into high economic food products. The flour of the fruit contains sufficient proximates for supporting the daily intake of nutrition. The flour from mangrove fruit presents an antioxidant and anti-diabetes to animal rat models. The innovation in processing mangroves resulted in several edible food products from snacks such as biscuits or crackers; drinks such as kombucha, tea, and coffee; and even synthetic staple food such as rice. The food products derived from mangrove fruit show merit in animal rat models. Unfortunately, the study on the introduction of mangrove fruit to functional food is limited to only some species, such as *B. gymnorrhiza, R. mucronata*, *S. caseolaris*, and *A. marina*, though around 27 species are confirmed traditionally and pharmacologically for their ethnomedicinal properties. This limitation opens challenges to identifying the possibility of developing the other mangrove species into functional foods. The mangrove plant is also considered the traditional medicine in the Middle East, and *A. marina* is the dominant mangrove species in the region. The study of phytochemicals from the fruit of mangrove is limited, and the development of fruit into functional food is even more limited.

Thus, the mangrove fruits can be used as a functional food with health impacts. Some local communities have utilized mangrove fruit in the form of homemade food products for traditional medicine since time immemorial. The further development of food based on mangrove fruit still requires a long journey, such as the economic and market study as well the cultivation strategy. The introduction of food derived from mangrove fruit to a vast community may also improve the understanding of the importance of mangrove forests.

## Figures and Tables

**Table 2:** The secondary metabolites from the fruit of mangroves and their bioactivity.

Mangrove species	Solvent	Compound	Bioactivity	Ref
*Xylocarpus granatum*	Water-ethanol	Gedunin (**1**)	Anticancer	[70]
*X. granatum*	Ethanol	Xyloccensin K (**2**)	Nt	[71]
		6-acetoxycedrodorin (**3**)	Nt	
		Xyloccensin W (**4**)	Nt	
*X. granatum*	Ethanol	3-deacetyl xyloccensin M (**5**)	Nt	[72]
		3-deacetyl xyloccensin N (**6**)	Nt	
*X. granatum*	Ethanol	Xyloccensin X_1_ (**7**)	Nt	[73]
		Xyloccensin X_2_ (**8**)	Nt	
*X. granatum*	Ethanol	Xyloccensin Y (**9**)	-	[74]
		Xyloccensin Z1 (**10**)	-	
		Xyloccensin Z2 (**11**)	-	
*X. granatum*	Ethanol	Xylogranatin A (**12**)	Nt	[75]
		Xylogranatin B (**13**)	Nt	
		Xylogranatin C (**14**)	Nt	
		Xylogranatin D (**15**)	Nt	
*X. granatum*	Ethanol	Xylogranatin E (**16**)	Nt	[76]
*X. granatum*	Ethanol	Xylocarpin A (**17**)	Nt	[69]
		Xylocarpin B (**18**)	Nt	
		Xylocarpin C (**19**)	Nt	
		Xylocarpin D (**20**)	Nt	
		Xylocarpin E (**21**)	Nt	
		6-dehydroxyxylocarpin D (**22**)	Nt	
		Xylocarpin F (**23**)	Nt	
		Xylocarpin G (**24**)	Nt	
		Xylocarpin H (**25**)	Nt	
		Xylocarpin I (**26**)	Nt	
*X. granatum*	Ethanol	Xylogranatinin (**27**)	Nt	[77]
*X. granatum*	Ethanol	Protoxylocarpin A (**28**)	Antitumor	[78]
		Protoxylocarpin B (**29**)	Antitumor	
		Protoxylocarpin C (**30**)	Antitumor	
		Protoxylocarpin D (**31**)	Antitumor	
		Protoxylocarpin E (**32**)	Antitumor	
		Xylocarpin J (**33**)	Antitumor	
		Xylocarpin K (**34**)	-	
		Xyloccensin M (**35**)	Antitumor	
*X. granatum*	Ethanol	Butyrospermol 3*β-O*-palmitate (**36**)	-	[79]
		Butyrospermol 3β*-O*-oleate (**37**)	-	
		Butyrospermol 3β*-O*-stearate (**38**)	-	
		Butyrospermol 3β*-O*-linoleate (**39**)	-	
*A. marina*	Ethanol	Maricaffeolylide A (**40**)	Antioxidant	[80]
		Maricyclohexene A (**41**)	Antioxidant	
*A. marina*	Ethanol-CH_2_Cl_2_	Marinoid F (**42**)	Antioxidant	[81]
		Marinoid G (**43**)	Antioxidant	
		Marinoid H (**44**)	Antioxidant	
		Marinoid I (**45**)	Antioxidant	
*A. marina*	Ethanol-CH_2_Cl_2_	Marinoid J (**46**)	Antioxidant	[82]
		Marinoid K (**47**)	Antioxidant	
		Marinoid L (**48**)	Antioxidant	
		Marinoid M (**49**)	Antioxidant	
*X. granatum*	Ethanol	Granatione (**50**)	Nt	[83]
		Xylocarpin L (**51**)	Nt	
*C. tagal*	Hexane-CH_2_Cl_2_	Cereotagaloperoxide (**52**)	-	[84]
		Cereotagalol A (**53**)	-	
		Cereotagalol B (**54**)	-	
		Isofouquierol (**55**)	-	
		Fouquierol (**56**)	-	
		3β*-E*-feruloylbetulinic acid (**57**)	Anticancer	
*S. apetala*	Ethanol	Sonneradon A (**58**)	Nematode’s life expansion	[85]
		Sonneradon B (**59**)	Nematode’s life expansion	
		Sonneradon C (**60**)	-	
		Sonneradon D (**61**)	Nematode’s life expansion	
		Ranuncoside (**62**)	-	
		Apigenin (**63**)	Nematode’s life expansion	
		Luteoline (**64**)	-	
		6-*O*-galloyl-*d*-glucopyranose (**65**)	-	
		*O-B*-(6-*O*-galloyl)-glucopyranoside (**66**)	-	
		2-ethylhexyl phthalate (**67**)	-	
		Methyl gallate (**68**)	-	
		Methyl 4-*O*-methylgallate (**69**)	-	
		4-*O*-methylgallic acid (**70**)	-	
		4-methoxybenzoic acid (**71**)	-	
		3,4-dihydrobenzoic acid (**72**)	-	
		Bibutyl malate (**73**)	-	
		Dimethyl malate (**74**)	Nematode’s life expansion	
		Bibutyl malate (**75**)	-	
		Ethylmethyl malate (**76**)	-	
		2-butenedioic acid (**77**)	-	
		3-hydroxy-4-oxobutanoic acid (**78**)	-	
		Butylmethyl malate (**79**)	Nematode’s life expansion	
*X. granatum*	Acetonitrile	Piscidinol G (**80**)	Nt	[86]
		Xylogranation D (**81**)	Nt	
		Spicatin (**82**)	Nt	
		Xylogranatin C (**83**)	Nt	
		Xyloccensin V (**84**)	Nt	
		Proceranolide (**85**)	Nt	
		Xylomexicanin D (**86**)	Nt	
		Sapelin E acetate (**87**)	Nt	
		Xylomexicanin A (**88**)	Nt	
		Grandifoliolenone (**89**)	Nt	
		Odoratone (**90**)	Nt	

Nt = not tested, (-) = no activity/no information.

**Table 3:** The metabolites isolated from the seed of mangrove and their bioactivities.

Mangrove species	Solvent	Compound	Bioactivity	Ref
*X. granatum*	Light petroleum	Xyloccensin I (**91**)	-	[89]
		Xyloccensin J (**92**)	-	
*X. moluccensis*	Ethanol	Godavarin A (**93**)	Insecticidal and antifeedant	[90]
		Godavarin B (**94**)	-	
		Godavarin C (**95**)	-	
		Godavarin D (**96**)	Insecticidal and antifeedant	
		Godavarin E (**97**)	-	
		Godavarin F (**98**)	-	
		Godavarin G (**99**)	-	
		Xyloccensin L (**100**)	-	
		Godavarin H (**101**)	-	
		Godavarin I (**102**)	-	
		Godavarin J (**103**)	Insecticidal and antifeedant	
		Xyloccensin P (**104**)	-	
		Xyloccensin Q (**105**)	-	
		Angustidienolide (**106**)	Insecticidal and antifeedant	
		6-deoxy-3detigloyl-swietenine acetate (**107**)	Insecticidal and antifeedant	
		Fissinolide (**108**)	Insecticidal and antifeedant	
		Methyl 3β-acetoxy-1-oxomeliaca-8(9),14-dienoate (**109**)	Insecticidal and antifeedant	
*X. granatum*	Ethanol	30α-hydroxyl xylogranatin A (**110**)	Nt	[91]
		Xylogranatin E2 (**111**)	Nt	
*X granatum*	Ethanol	Thaigranatin A (**112**)	-	[92]
		Thaigranatin B (**113**)	-	
		Thaigranatin C (**114**)	-	
		Thaigranatin D (**115**)	-	
		Thaigranatin E (**116**)	-	
		Granatumin L (**117**)	Anti-HIV	
*X. granatum*	Ethanol	Xylomexicanin A (**118**)	Antitumor	[93]
		Xylomexicanin B (**119**)	-	
*X. moluccensis*	Ethanol	Moluccensin R (**120**)	Antifeedant	[94]
		Moluccensin S (**121**)	-	
		6-hydroxymexicanolide (**122**)	Antifeedant	
		2-hydroxyfissinoide (**123**)	Antifeedant	
		Moluccensin T (**124**)	-	
		Moluccensin U (**125**)	-	
		Moluccensin V (**126**)	-	
		Moluccensin W (**127**)	-	
		Moluccensin X (**128**)	-	
		Moluccensin Y (**129**)	-	
*X. moluccensis*	Ethanol	Krishnolide A (**130**)	Anti-HIV	[95]
		Krishnolide B (**131**)	-	
		Krishnolide C (**132**)	-	
		Krishnolide D (**133**)	-	
*X. moluccensis*	Ethanol	Xylomolin A1 (**134**)	-	[96]
		Xylomolin A2 (**135**)	-	
		Xylomolin A3 (**136**)	-	
		Xylomolin A4 (**137**)	-	
		Xylomolin A5 (**138**)	-	
		Xylomolin A6 (**139**)	-	
		Xylomolin A7 (**140**)	-	
		Xylomolin B1 (**141**)	-	
		Xylomolin B2 (**142**)	-	
		Xylomolin C1 (**143**)	-	
		Xylomolin C2 (**144**)	-	
		Xylomolin D (**145**)	-	
		Xylomolin E (**146**)	-	
		Xylomolin F (**147**)	-	
		Xylomolin G1 (**148**)	-	
		Xylomolin G2 (**149**)	-	
		Xylomolin G3 (**150**)	-	
		Xylomolin G4 (**151**)	-	
		Xylomolin G5 (**152**)	-	
		Xylomolin H (**153**)	-	
		Xylomolin I (**154**)	-	
		Xylomolin J1 (**155**)	-	
		Xylomolin J2 (**156**)	Anticancer	
		Xylomolin K1 (**157**)	-	
		Xylomolin K2 (**158**)	-	
		Xylomolin L1 (**159**)	-	
		Xylomolin L2 (**160**)	-	
		Xylomolin M (**161**)	-	
		Xylomolin N (**162**)	-	
*X. moluccensis*	Ethanol	Thaixylomolin O (**163**)	-	[97]
		Thaixylomolin P (**164**)	-	
		Thaixylomolin Q (**165**)	-	
		Thaixylomolin R (**166**)	-	
*X. granatum*	Ethanol	Granatumin M (**167**)	-	[98]
		Granatumin N (**168**)	-	
		Granatumin O (**169**)	-	
		Granatumin P (**170**)	-	
		Granatumin Q (**171**)	-	
		Granatumin R (**172**)	-	
		Granatumin S (**173**)	-	
		Granatumin T (**174**)	-	
		Granatumin U (**175**)	-	
*X. granatum*	Methanol	Sundarbanxylogranin A (**176**)	-	[99]
		Sundarbanxylogranin B (**177**)	Anti-HIV	
		Sundarbanxylogranin C (**178**)	-	
		Sundarbanxylogranin D (**179**)	-	
		Sundarbanxylogranin E (**180**)	-	
*X granatum*	Ethanol	Granatumin X (**181**)	-	[98]
		Krishnagranatinin A (**182**)	-	
		Krishnagranatinin B (**183**)	-	
		Krishnagranatinin C (**184**)	-	
		Krishnagranatinin D (**185**)	-	
		Krishnagranatinin E (**186**)	-	
		Krishnagranatinin F (**187**)	-	
		Krishnagranatinin G (**188**)	Inhibit NF-κB	
		Krishnagranatinin H (**189**)	Inhibit NF-κB	
		Krishnagranatinin I (**190**)	Inhibit NF-κB	
*X. granatum*	Ethanol	Granaxylocarpin A (**191**)	Anticancer	[100]
		Granaxylocarpin B (**192**)	Anticancer	
		Granaxylocarpin C (**193**)	-	
		Granaxylocarpin D (**194**)	-	
		Granaxylocarpin E (**195**)	-	
*X. granatum*	Ethanol	Thaixylogranin A (**196**)	Anticancer	[101]
		Thaixylogranin B (**197**)	Anticancer	
		Thaixylogranin C (**198**)	Anticancer	
		Thaixylogranin D (**199**)	Anticancer	
		Thaixylogranin E (**200**)	Anticancer	
		Thaixylogranin F (**201**)	Anticancer	
		Thaixylogranin G (**202**)	Anticancer	
		Thaixylogranin H (**203**)	Anticancer	
*X. moluccensis*	Ethanol	Trangmolin A (**204**)	-	[102]
		Trangmolin B (**205**)	-	
		Trangmolin C (**206**)	-	
		Trangmolin D (**207**)	-	
		Trangmolin E (**208**)	-	
		Trangmolin F (**209**)	-	
*X. moluccensis*	Ethanol	Thaixylomolin G (**210**)	-	[103]
		Thaixylomolin H (**211**)	-	
		Thaixylomolin I (**212**)	Anti-H1N1	
		Thaixylomolin J (**213**)	-	
		Thaixylomolin K (**214**)	Anti-H1N1	
		Thaixylomolin L (**215**)	-	
		Thaixylomolin M (**216**)	Anti-H1N1	
		Thaixylomolin N (**217**)	-	
		12-deacetylxyloccensin U (**218**)	-	
		2-*O*-acetyl-2-dehydroxy-12-deacetylxyloccensin (**219**)	-	
		6-*O*-acetyl-2a-hydroxymexicanolide (**220**)	-	
		6-*O*-acetyl-6-dehydroxymoluccensin T (**221**)	-	
*X. rumphii*	Methanol	Xylorumphiin E (**222**)	-	[104]
		Xylorumphiin F (**223**)	-	
		2-hydroxyxylorumphiin F (**224**)	Antiinflammatory	
		Xylorumphiin G (**225**)	Antiinflammatory	
		Xylorumphiin H (**226**)	-	
		Xylorumphiin I (**227**)	-	
		Xylorumphiin J (**228**)	-	

Nt = not tested, (-) = no activity/no information.

**Table 4:** Metabolites of endophytic fungus from mangrove fruit and their bioactive properties.

Mangrove species	Fungus species	Cultivation media	Compound	Bioactivity	Ref
*K. candel*	*Penicillium commune*	Rice substrate	Peniisocoumarin A (**229**)	-	[106]
			Peniisocoumarin B (**230**)	-	
			Peniisocoumarin C (**231**)	α-glucosidase inhibition	
			Peniisocoumarin D (**232**)	-	
			Peniisocoumarin E (**233**)	α-glucosidase inhibition	
			Peniisocoumarin F (**234**)	α-glucosidase inhibition	
			Peniisocoumarin G (**235**)	α-glucosidase inhibition	
			Peniisocoumarin H (**236**)	-	
			Peniisocoumarin I (**237**)	α-glucosidase inhibition	
			3-[-(*R*)-3,3-dichloro-2-hydroxypropyl]-8-hydroxy-6-methoxy-1*H*-isochromen-1-on1 (**238**)	α-glucosidase inhibition	
			Peniisocoumarin J (**239**)	α-glucosidase inhibition	
			(+)-6-methyl-citreoisocoumarin (**240**)	-	
			(+)-diaporthin (**241**)	-	
*S. caseolaris*	*Alternaria* sp.	Rice substrate	Altenusin derivative 1 (**242**)	-	[107]
			Altenusin derivative 2 (**243**)	α-glucosidase inhibition	
			Altenusin derivative 3 (**244**)	α-glucosidase inhibition	
			Altenusin derivative 4 (**245**)	α-glucosidase inhibition	
			Altenusin derivative 5 (**246**)	-	
			Talaroflavone (**247**)	α-glucosidase inhibition	
			Deoxyrubralactone (**248**)	-	
			Rubralactone (**249**)	α-glucosidase inhibition	
			2-OH-AOH (**250**)	α-glucosidase inhibition	
			Alternariol (**251**)	*α*-glucosidase inhibition	
			Alternariol methyl ether (**252**)	-	
*Acanthus ilicifolius*	*Epicoccum nigrum*	Wheat solid substrate	Racemix (±)-epicoccone C (**253**)	*α*-glucosidase inhibition	[108]
			Epicoccone D (**254**)	*α*-glucosidase inhibition	
			Epicoccone E (**255**)	*α*-glucosidase inhibition	
			Epicolactone A (**256**)	*α*-glucosidase inhibition	
			Epicolactone (**257**)	-	
			Flavimycins A (**258**)	*α*-glucosidase inhibition	
			Epicocconigrone A (**259**)	*α*-glucosidase inhibition	
			Epicoccolide B (**260**)	*α*-glucosidase inhibition	
*A. marina*	*Aspergillus versicolor*	White bean	Allantopyrone E (**261**)	Anticancer	[109]
*K. candel*	*Botryosphaeria* sp.	Rice substrate	Botryoisocoumarin A (**262**)	COX-2 inhibition	[110]
			Monocerin (**263**)	-	
			3-methyl-6,8-dihydroxyisocoumarin (**264**)	-	
			8-methoxymellein (**265**)	-	
			*Trans*-4-hydroxymellein (**266**)	-	
			5-hydroxy-7-methoxy-4,6-dimethyl phthalide (**267**)	-	
*K. obovata*	*Talaromyces amestolkiae*	Rice substrate	Talanaphthoquinone A (**268**)	Antioxidant	[111]
			Talanaphthoquinone B (**269**)	Antioxidant	
			Anhydrojavanicin (**270**)	Antioxidant	
			2,3-dihydro-5-hydroxy-4-hydroxymethyl-8-methoxy-2-methylnaphtho[1,2-b]furan-6,9-dione (**271**)	Antioxidant	
			Anhydrofusarubin (**272**)	Antioxidant	
			2-acetonyl-3-methyl-5-hydroxy-7-methoxy-naphthazarin (**273**)	Antioxidant	
			6-ethyl-2,7-dimethoxyjuglone (**274**)	Antioxidant	
			6-[1-(acetyloxy)ethyl]-5-hydroxy-2,7-dimethoxy1,4-naphthalenedione (**275**)	Antioxidant	
			5-hydroxy-6-(1-hydroxyethyl)-2,7-dimethoxy-1,4-naphthalenedione (**276**)	Antioxidant	
			Solaniol (**277**)	Antioxidant	
			Javanicin (**278**)	Antioxidant	
*Bruguiera* sp.	*Mycosphaerella* sp.	Rice substrate	Asperchalasine I (**279**)	α-glucosidase inhibitor, antioxidant	[112]
			Dibefurin B (**280**)	-	
			(*R*)-9-((*R*)-10-hydroxyethyl)-7,9-dihydroisobenzofuran-1-ol (**281**)	-	
			2-methoxycarbonyl-4,5,6-trihydroxy-3-methyl-benzaldehyde (**282**)	Antioxidant	
			Epicoccone B (**283**)	-	
			1,3-dihydro-5-methoxy-7-methylisobenzofuran (**284**)	Antioxidant	
			Paeciloside A (**285**)	-	
			Asperchalasine A (**286**)	α-glucosidase inhibitor, antioxidant	
			Aspochalasin I (**287**)	-	
*S. apetala*	*Pseudofusicoccum* sp.	Rice substrate	Acorenone C (**288**)	AChE inhibition	[113]
			Uracil (**289**)	-	
			Cyclo-(*L*-Pro-*L*-Tyr) (**290**)	-	
			*Bis*-(2-ethylhexyl) terephthalate (**291**)	-	
			4-hydroxybenzaldehyde (**292**)	-	
			2-phenylethanol (**293**)	-	
			4-hydroxyphenethyl alcohol (**294**)	-	
			Estigmast-4-en-6*β*-ol-3-ona (**295**)	-	
			Ergosterol (**296**)	NO production inhibition; anticancer	
			Ergosterol peroxide (**297**)	-	
			Cerevisterol (**298**)	-	
*Aegiceras corniculatum*	*Alternaria* sp.	Potato dextrose broth	Alterporriol K (**299**)	Anticancer	[114]
			Alterporriol L (**300**)	Anticancer	
			Alterporriol M (**301**)	-	
			Physcion (**302**)	-	
			Marcrospin (**303**)	-	
			Dactylariol (**304**)	-	
			Tetrahydroaltersolanol B (**305**)	-	

Nt = not tested, (-) = no activity/no information.

**Table 5:** Patents in the mangrove fruit transformation in food products.

Innovation	Patent No	Ref
Processing the mangrove fruit	CN10314178B	[154]
The fruit is soaked in saline water and then cleaned after the peel is softened. The fruit is sprayed with white wine and then soaked in hot water at 70–90 °C. The peel is removed afterward. The fruit is soaked in warm water at 30–40 °C then dried, sterilized, and packaged.		
Processing the mangrove fruit	CN10460543A	[155]
The fruit is cleaned and peeled then ground using an ultra-micro grinder to make a fine powder. The powder is mixed with water, homogenized, and enzymolized by protease. The filtrate is removed and vacuumed to decolorize from dark green to white. The slurry is dry, sterilized, and packaged.		
Synthetic rice from mangrove fruit starch	CN105166628	[153]
Brief description: mangrove fruit flour, glutinous rice flour, cornstarch, converted starch, and konjac flour are mixed and then pre-gelatinized. The pre-gelatinized dough is granulated, steamed, dried, then polished to make synthetic rice.		
Antitumor from mangrove fruit particle	CN106107961	[156]
Brief description: the fruit is cleaned and then mixed with water to make liquor. White sugar is added and centrifuged. The slurry is mixed with methylcellulose, glyceryl monostearate, and banana juice. The mixture is pelleted, dried, and sterilized.		
Wine from mangrove fruit	CN107557227	[150]
Brief description: mangrove fruit is soaked with limewash for 12–24 h at 50–80 °C, then cleaned. The yeast is added to the paste and fermented at 34–36 °C and the filtrate is collected afterward. The filtrate is fermented for 10–20 days to produce wine.		
Alcohol from mangrove fruit for removing blood stasis	CN107574079	[152]
Brief description: mangrove fruit is soaked in limewash, cleaned, then mixed with glutinous rice to make a paste. The yeast is added and fermented to produce alcohol.		
Teabag from mangrove fruit	CN107593976	[151]
Brief description: the mangrove fruit is soaked with limewash then cleaned and dried. The biomass is mixed with the fresh flower and then dried together as tea.		
Tea to decrease the blood-pressure from mangrove fruit	CN107549412A	[157]
Brief description: mangrove fruit is soaked in limewash then cleaned and dried. Thorn apple is soaked in an alcoholic solution and then dried. The dried thorn apple and mangrove fruit are ground and mixed. The powder is ready as tea.		
Chocolate from mangrove fruit Avicennia marina	CN103141648A	[147]
Brief description: the mangrove fruit is cleaned and mixed with liquor. The pulp is mixed with cocoa powder, whole milk powder, and skimmed milk powder to form chocolate.		
Chocolate from mangrove fruit	CN101496549A	[158]
The fruit is soaked in boiling water followed by cold water, then the peel is removed. The fruit is soaked in boiling water several times. The fruit is dried, crushed, and ground to make powder. The powder is mixed with milk powder, cocoa butter, sugar, and cocoa powder. The mixture is mixed, molded, and packed.		
Flavoring food from mangrove fruit	CN103750209A	[148]
Brief description: the mangrove fruit is mixed with vegetable protein hydrolase. The enzymolysis pulp is mixed with xanthan, acesulfame, and sugar, and then sterilized. The mixture is ready for flavoring food.		
Flavoring sauce from mangrove fruit	CN104323217A	[149]
Brief description: the mangrove fruit is boiled and then mixed with pepper powder, Chinese cassia, and cardamom. The mixture is then boiled and packaged.		
Syrup from mangrove fruit	CN101268796A	[159]
Brief description: the mangrove fruit is sterilized at 95–100 °C for 5–6 minutes then cooled. The fruit is soaked with CaCl_2_ and then cleaned. The honey containing sugar (1:30) is added followed by citric acid. The fruit is boiled and then packed into a can.		

**Table 6:** Several studies of bioactive compounds from the mangrove forest in the Middle East.

Location	Sample type	Experiment	Activity	Ref
Jazan, Red Sea coast of Saudi Arabia	Fruit of *A. marina*	Ethanol extract	Antibacterial against *P. aeruginosa*, *B. subtilis*, *S. aureus*, *E.coli* Antifungal against *A. fumigatus*, *C. albicans*	[58]
	Seed of *A. marina*	Ethanol extract	Antifungal activities against *A. fumigatus*	
	Root of *A. marina*	Chloroform extract	Antibacterial against *S. aureus*, *E. coli* Antifungal against *A. fumigatus*	
	Leaves of *A. marina*	Ethyl acetate extract	Antibacterial against *S. aureus*, *E. coli*	
Safaga, Red Sea coast of Egypt	Seed of *A. marina*	Chloroform and ethanol extract	Antibacterial against *P. aeruginosa*, *V. fluvialis*, *V. vulnificus*, *S. fecalis*, *E. coli*, *B. subtilis*, *S. aureus*	[218]
	Leaves, stems, and roots of *A. marina*	Chloroform extract	Antibacterial against *P. aeruginosa*, *V. fluvialis*, *V. vulnificus*, *S. fecalis*, *E. coli*, *B. subtilis*, *S. aureus*	
	Leaves, stems, and roots of *A. marina*	Ethanol extract	Antibacterial against *V. fluvialis*, *V. vulnificus*	
Red Sea coast of Egypt	Sediment sample from mangrove forest	Actinomycetes isolation and extraction	Antimicrobial against *B. subtilis*, *E. coli*, *S. aureus*, *P. aeruginosa*, *C. albicans*.	[219]
Red Sea coast of Saudi Arabia	Decayed leaves of *A. marina*	Black yeast *Hortaea werneckii*	Antimicrobial against pathogen *S. aureus*, *Campylobacter jejuni*, and *S. typhimurium*.	[220]
